# Single-cell RNA sequencing of human epidermis identifies Lunatic fringe as a novel regulator of the stem cell compartment

**DOI:** 10.1016/j.stemcr.2023.09.007

**Published:** 2023-10-12

**Authors:** Victor Augusti Negri, Blaise Louis, Sebastiaan Zijl, Clarisse Ganier, Christina Philippeos, Shahnawaz Ali, Gary Reynolds, Muzlifah Haniffa, Fiona M. Watt

**Affiliations:** 1 King’s College London Centre for Stem Cells and Regenerative Medicine, Guy’s Hospital, London, UK; 2 Biosciences Institute, Newcastle University, Newcastle upon Tyne, UK; 3 Wellcome Sanger Institute, Wellcome Genome Campus, Cambridge, UK; 4 Directors’ Research Unit, European Molecular Biology Laboratory, Heidelberg, Germany

**Keywords:** Epidermis, stem cell, Notch pathway, DLL1, FRINGE, scRNAseq

## Abstract

Single-cell RNA sequencing (scRNA-seq) of human skin provides a tool for validating observations from *in vitro* experimental models. By analyzing a published dataset of healthy adult epidermis, we confirm that the basal epidermal layer is heterogeneous, and three subpopulations of non-dividing cells can be distinguished. We show that Delta-like ligand 1 (DLL1) is expressed in a subset of basal cells previously identified as stem cells in cultured human keratinocytes and map the distribution of other Notch ligands and receptors to specific epidermal cell compartments. Although DLL1 is expressed at low levels, it is expressed in the same cell state as the Notch regulator, Lunatic -fringe (LFNG, O-fucosylpeptide 3-beta-N-acetylglucosaminyltransferase). Overexpression of LFNG amplifies the effects of DLL1 in cultured keratinocytes, increasing proliferation and colony-forming ability. We conclude that using scRNA-seq resources from healthy human skin not only validates previous experimental data but allows formulation of testable new hypotheses.

## Highlights

• The basal compartment of human epidermis is heterogeneous
• Notch signaling plays a role in stem cell maintenance and differentiation
• LFNG amplifies DLL1 signaling in stem cells

## Teaser

In this study, Negri and colleagues re-analyze published single-cell RNA sequencing data from adult human epidermis to validate previous conclusions from cultured keratinocytes and to generate new mechanistic hypotheses. They show that the basal layer is heterogeneous, and co-expression of DLL1 and LFNG in stem cells amplifies the effect of DLL1 in stem cell maintenance.

## Introduction

The outermost skin layer, the epidermis, comprises a stratified squamous epithelium, the interfollicular epidermis (IFE), and adnexal appendages (Watt, 2014). IFE proliferation takes place mainly in the basal layer, where the stem cells reside, and on initiation of terminal differentiation, cells move through the suprabasal layers, finally differentiating into corneocytes.

One of the pathways that controls the balance between IFE differentiation and proliferation is the Notch pathway. Notch signaling induces growth arrest and terminal differentiation of cultured keratinocytes (Lowell et al., 2000; Watt et al., 2008). Activation of Notch targets is observed in the suprabasal layers of mouse epidermis (Estrach et al., 2006; Nguyen et al., 2006) and in cultured keratinocytes. Notch activity occurs predominantly in cells committed to, or initiating, terminal differentiation (Rangarajan et al., 2001; Blanpain et al., 2006; Negri et al., 2019). Conditional ablation of *NOTCH1* in mice increases proliferation and decreases differentiation.

Three Notch ligands are found in human epidermis: *DLL1*, *JAG1*, and *JAG2* (Watt et al., 2008). *DLL1* is expressed in clusters of cells in the basal layer of human and mouse fetal epidermis (Rangarajan et al., 2001; Estrach et al., 2008). In cultured human keratinocytes, DLL1 plays a role in the stem cell compartment, inhibiting Notch signaling via *cis* inhibition (Lowell et al., 2000; Negri et al., 2019).

Notch signal modulation can occur at different levels, including glycosylation of ligands and receptors, ubiquitylation, endocytosis, and trafficking (Irvine, 2008; Stanley and Okajima, 2010). Fringe proteins are N-acetylglycosyltransferases (Irvine, 2008; LeBon et al., 2014) that transfer N-acetylglucosamine to O-fucose residues in Notch EGF repeats in the Golgi complex (Irvine, 2008). These enzymes can change the Notch extracellular domain (NECD) affinity for Notch ligands in the same cell or an adjacent cell. Lunatic fringe (LFNG) and Manic fringe (MFNG) increase Notch affinity for DLL1 and inhibit JAG receiving signals (LeBon et al., 2014). Radical fringe (RFNG) can increase the affinity for both ligands (Irvine, 2008).

*In vitro* studies of human keratinocytes have identified stem cell markers, using colony formation as a quantitative readout of stem cell number (Lowell et al., 2000; Tan et al., 2013). These studies point to the existence of multiple subpopulations of cells in the epidermal basal layer, a conclusion supported by scRNA-seq of keratinocytes isolated directly from healthy adult human skin (Cheng et al., 2018; Wang et al., 2020; Reynolds et al., 2021; Negri and Watt, 2022). In the present study, we have analyzed the scRNA-seq data in more depth, to gain insights into the role of DLL1 in the stem cell compartment and the different stages of differentiation at which the Notch pathway is active.

## Results

### Mapping known epidermal markers to cell clusters identified by scRNA-seq

**Figure 1:**
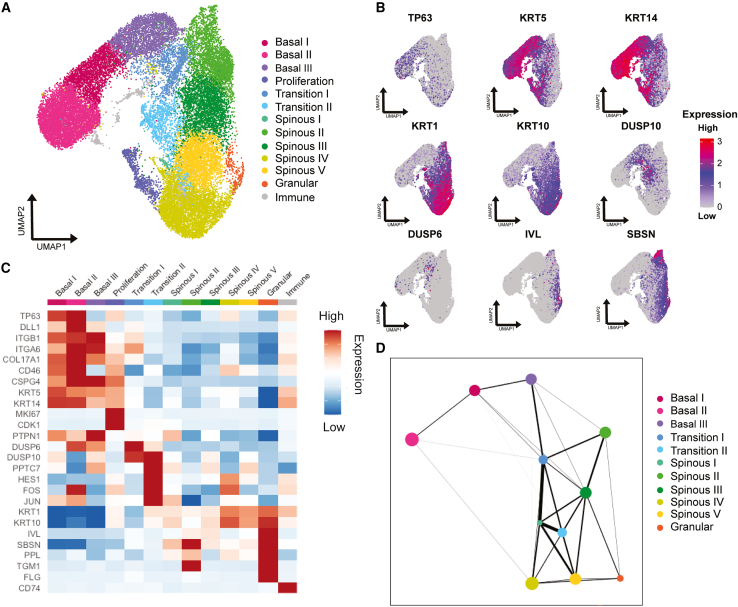
Transcriptional heterogeneity of human epidermal keratinocytes *in vivo* (A and B) UMAP plots showing 13 distinct cell states found in human IFE (five donors, 27,138 cells) (A) and expression of known markers (B). (C) Heatmap of averaged expression of genes differentially expressed in distinct cell states (log fold change). (D) PAGA connectivity plot after removing the *Proliferation* and *Immune* states. Edge thickness corresponds to strength of connection between nodes. Node size reflects respective cluster (cell state) size. See also Figure S1.

We explored Notch signaling using an unbiased approach with an scRNA-seq dataset of human IFE keratinocytes from five healthy adult donors (Reynolds et al., 2021; Negri and Watt, 2022). After quality control, clustering, and dimensionality reduction, we identified 13 distinct transcriptomic profiles (Figures 1A–1C and S1). All clusters were found in all five samples sequenced (Figure S1A). The five most highly expressed genes in each population are presented in Figure S1B.

Based on expression of basal layer markers such as *KRT5* and *14*, we assigned four clusters to the basal layer: *Basal I, II, III*, and *Proliferation*, the latter expressing high levels of cyclin-dependent kinase 1 (*CDK1*) and Marker of Proliferation Ki-67 (*MKI67*) (Figure 1C). The classic cell surface markers used to enrich for clonogenic human keratinocytes are integrins α2β1, α3β1, and α6β4. *ITGA3* and *ITGA6* were most abundant in *Basal II*, while *ITGA2* and *ITGB1* were most abundant in *Basal III*. *CD46*, identified as a marker of Dll1+ stem cells (Tan et al., 2013), was most highly expressed in *Basal II*, as was *DLL1* (Figure 1C). *COL17A1*, *MT2A*, *CXCL24*, and *POSTN* were also expressed most highly in *Basal II* (Figure 1C). *CAV1* and *CAV2* are markers of *DLL1* high stem cells in culture (Tan et al., 2013), and *CAV1* was one of the most highly expressed cells in *Basal I* (Figure S1B).

We assigned two clusters as *Transition* based on expression of pro-commitment genes such as the protein phosphatases DUSP6 and DUSP10 (Mishra et al., 2017). Consistent with antibody labeling in human epidermis (Mishra et al., 2017), expression of *DUSP10* was more widespread than *DUSP6* (i.e., upregulated in *Transition I* and *II*) (Figure 1B).

Five clusters were ascribed to the first suprabasal (spinous) cell layers, based on expression of *KRT1* and *KRT10*. Granular cells were assigned based on *FLG* expression, the low number of cells in this cluster probably reflecting the difficulty in isolating cells from the uppermost IFE layers (Cheng et al., 2018).

We also identified a population of keratinocytes categorized as *Immune* based on co-expression of basal (*KRT5*, *KRT14*) and suprabasal (*KRT10*) markers and the macrophage migration inhibitory factor receptor *CD74*. A cluster of keratinocytes expressing *CD74* and other immune signature genes (Figure S1B) has been reported previously (Cheng et al., 2018). Immunostaining of human epidermis revealed scattered CD74^+^ cells with a dendritic morphology in the basal and suprabasal cell layers (Figure S1). CD74 is expressed by dendritic cells (Su et al., 2017), and we speculate that the immune cluster in the scRNA-seq dataset comprises dendritic cells that have ingested keratinocyte RNA via trogocytosis (Zhao et al., 2022).

We used partition-based approximate graph abstraction (PAGA) to analyze connections between the different cell states (Figure 1D). We omitted *Immune* because it does not appear to be part of the normal differentiation trajectory and *Proliferation* because proliferation and differentiation are under separate control (e.g., Mishra et al., 2017). The strongest inferred trajectory was cells moving through *Basal II* to *Basal I* to *Basal III* and then into *Transition I*. This supports the definition of *Basal II* as the most stem-like state based on *DLL1* expression. The *Transition* states showed strong connectivity with the *Spinous* states and converged on *Granular*. The existence of distinct trajectories in the spinous cell clusters was previously reported (Reynolds et al., 2021).

### Notch pathway gene expression in epidermal cell populations

**Figure 2:**
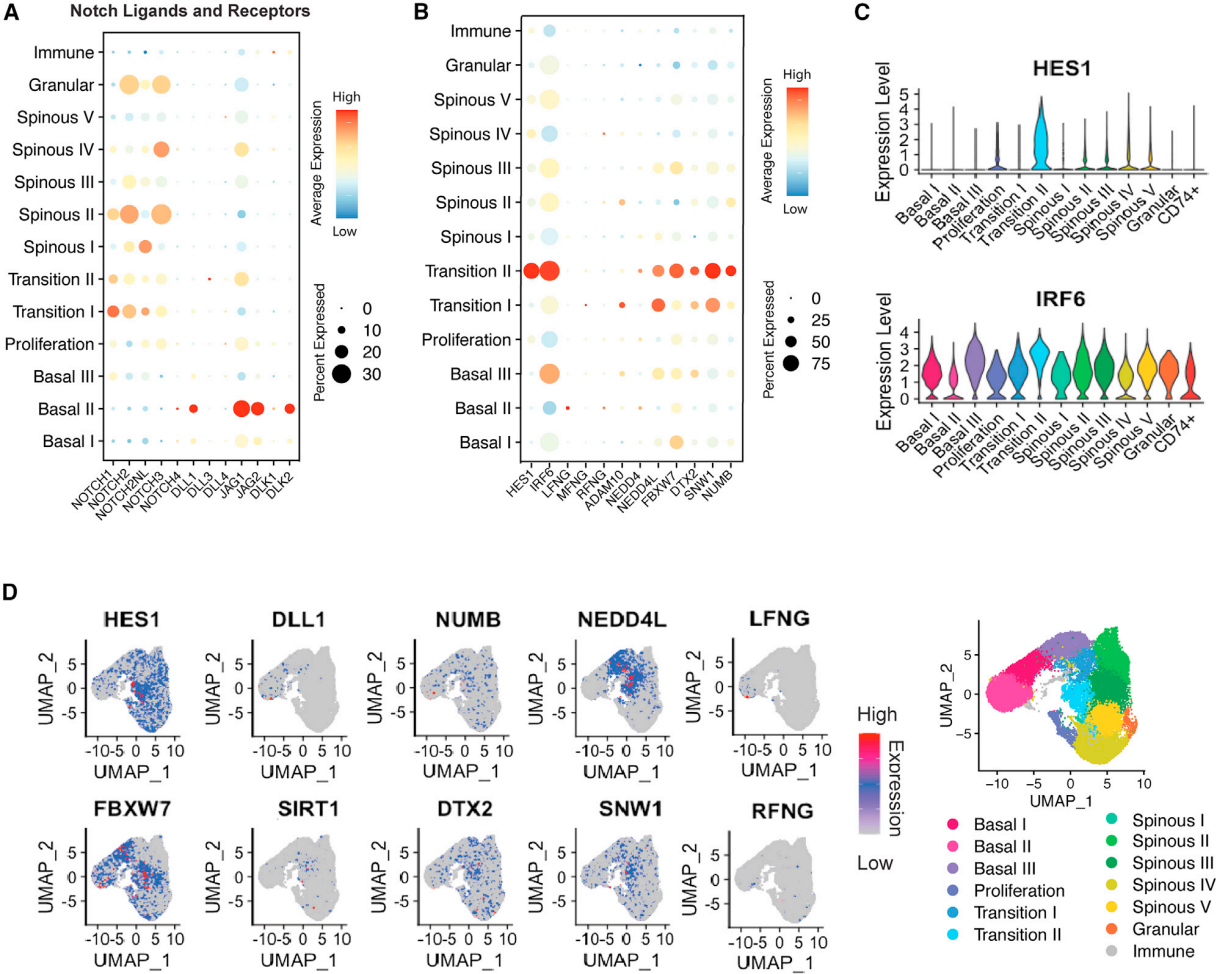
Expression of Notch-related genes in keratinocyte clusters (A and B) Dot plots showing Notch signaling pathway ligands and receptors (A) and pathway targets and regulators (B). (C) Violin plots of *HES1* and *IRF6* expression. (D) UMAP plots of expression distribution of distinct Notch pathway components; location of the 13 clusters (Figure 1A) is included for comparison. See also Figures S2 and S3.

Next, we investigated which cells express different Notch ligands and receptors (Figure 2). In addition to *DLL1*, *JAG1* and *JAG2* were predominantly expressed in *Basal II* (Figure 2A). The levels of mRNAs for *DLL3* and *DLL4* were almost undetectable (Negri et al., 2019). We also found low levels of Delta-Like Non-Canonical Notch Ligand 1 and 2 (*DLK1* and *DLK2*) in *Basal II* (Figure 2A). In contrast to Notch ligands, Notch receptors were most abundant in the transition, spinous, and granular layer clusters. *NOTCH1* was most abundant in *Transition I*, while *NOTCH2* and *NOTCH3* were more highly expressed in *Spinous* and *Granular*. *NOTCH4* was almost undetectable (Negri et al., 2019).

We also analyzed expression of known Notch targets such as *HES1* and *IRF6* (Figures 2B–2D and S2). The highest expression of *HES1* was in *Transition II*, whereas *IRF6* was more widely expressed (Figures 2B and 2C). This supports a role of Notch signaling in commitment to differentiation (Negri et al., 2019). The enrichment of other pathways related to keratinocyte terminal differentiation and AP1 factors in *Transition II* suggests that this population comprises the cluster most committed to differentiation (Mishra et al., 2017) (Figures S2B and S2C).

Genes encoding proteins that cleave Notch receptors at the plasma membrane to release the Notch intracellular domain (NICD) include *ADAM17*, *ADAM10*, and *PSEN1*, all of which were abundant in *Transition I* and *II* (Figure S3A). In contrast, gamma-secretase subunit *APH1A* was most abundant in *Basal II* (Figure S3A).

The Nedd4 family member of HECT domain E3 ubiquitin ligases NEDD4 (neural precursor cell-expressed developmentally downregulated four/NEDD-1) and NEDD4L (neural precursor cell-expressed developmentally downregulated four-like/NEDD-2) direct Notch receptors to the lysosomes for degradation and are negative regulators of the Notch pathway (Kovall et al., 2017). *NEDD4L* was more abundant than *NEDD4* and was a marker for both *Transition I* and *II* (Figure S3D).

The E3 ubiquitin ligase FBXW7 or FBW7 (F Box and WD Repeat Domain Containing 7) binds directly to NICD in the nucleus, leading to its proteasomal degradation and termination of Notch signaling (Kourtis et al., 2015). We observed particularly high expression of *FBXW7* in *Transition II* (Figures 2B and 2D), the same cluster with the highest levels of the histone deacetylase Sirtuin-1 (SIRT1) (Figures 2B and 2D), which can associate with NICD and counteract the stabilizing effect of acetylation, modulating Notch activity amplitude and duration (Guarani et al., 2011).

Several post-translational modifiers of the Notch pathway exhibited differential expression. *FURIN* was the most abundant gene in *Transition I*, while *POGLUT1* was prominent in *Transition II*. *POGLUT1* was also upregulated in *Basal II*, as were *RFNG* and *LFNG*. *MFNG* was expressed at very low levels (Figures 2B, 2D, and S3B).

In conclusion, the scRNA-seq data support the experimental evidence that Notch signaling regulates the onset of terminal differentiation, with many mediators of the pathway being upregulated in the *Transition I* and *II* clusters. These findings further emphasize that the stem cell marker DLL1 is expressed at lower levels than other Notch ligands, raising the question of how it plays such a key role in the epidermal basal layer.

### Expression of Fringe proteins in the epidermis and cultured keratinocytes

**Figure 3:**
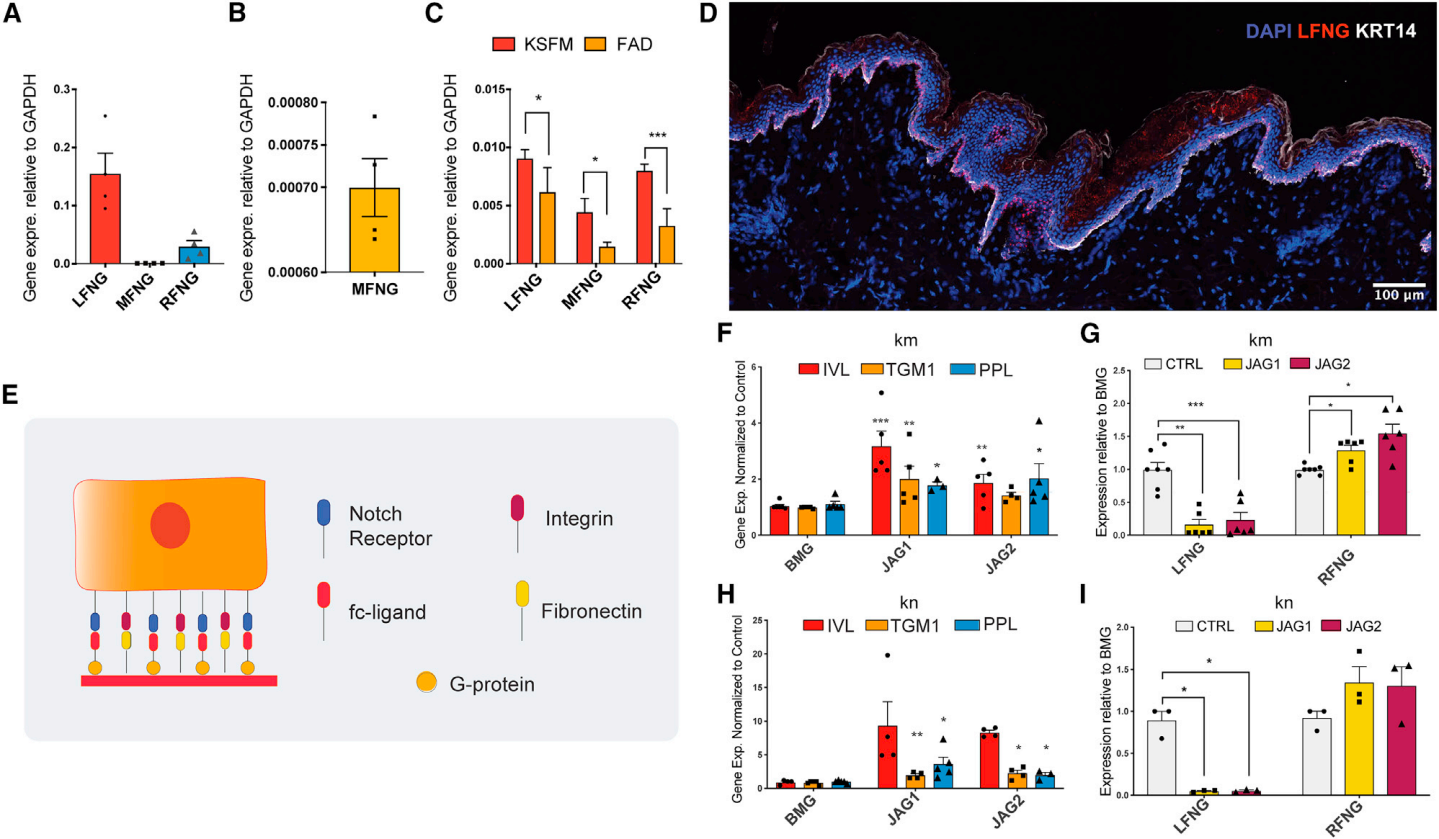
Differential expression of Fringe genes (A–C) Expression of Fringe genes in human epidermis (A and B) and cultured keratinocytes using qPCR. (C) Average fold change in mRNA abundance (normalized to expression of *RPS18*, *GAPDH*, and *TBP*) compared to control condition. (A–C) Error bars represent standard deviation. Two-tailed, unpaired Student’s t test. ^∗^p < 0.05, ^∗∗∗^p < 0.001. N = 4 samples. (D) smFISH of adult human skin for *LFNG* (red), counterstained with DAPI (blue) and anti-KRT14 (white). Scale bar, 200 μm. (E) Schematic of experimental setup for (F)–(I). (F–I) Expression of differentiation markers (F and H) and *LFNG* and *RFNG* (G and I) in two strains of keratinocytes (KM: km and KN: kn) exposed to recombinant Jagged fc-ligands. Bars represent average fold change compared to control. Each data point is a separate sample. One-way ANOVA with Holm Sidak’s multiple comparisons test. ^∗^p < 0.05, ^∗^p < 0.01, ^∗∗∗^p < 0.001. N = 3.

Consistent with the scRNA-seq data (Figure 2B), *LFNG* was more abundant than *RFNG*, and *MFNG* was expressed at very low levels in adult epidermis (Figures 3A and 3B). The same relative expression was observed in human keratinocytes cultured in low-calcium KSFM (keratinocyte serum free medium) medium or on feeders in standard calcium FAD medium (Figure 3C). *MFNG* expression was higher *in vitro* than *in vivo* (Figure 3C). There was higher expression of all *FNG* genes in KSFM than FAD medium, consistent with Fringe expression being more abundant in basal than differentiated cells. Single-molecule fluorescence *in situ* hybridization (smFISH) of *LFNG* transcripts in human epidermis confirmed expression of *LFNG* in basal layer keratinocytes, but it did not distinguish between particular subpopulations of cells (Figure 3D).

We next investigated how *LFNG* and *RFNG* expression was affected when primary keratinocytes (strains km, kn) were exposed to Jagged ligands. We seeded cells on fibronectin in the presence of recombinant Jagged1, Jagged2 or, as a control, beta2 microglobulin (Figure 3E; Negri et al., 2019). Exposing keratinocytes to Jagged1 or Jagged2 led to a significant upregulation of the terminal differentiation marker, *IVL*, with smaller effects on two other differentiation markers, *TGM1* and *PPL* (Figures 3F and 3H). *LFNG* expression was significantly reduced by Jagged1 and Jagged2, whereas *RFNG* was either unaffected or slightly increased (Figures 3G and 3I).

We conclude that in cultured and uncultured human epidermis, *LFNG* is the most abundant Fringe gene and is expressed by basal keratinocytes. When keratinocytes are stimulated to differentiate by exposure to Jagged ligands, expression of *LFNG* is selectively downregulated.

### Overexpression of LFNG increases proliferation and reduces differentiation

**Figure 4:**
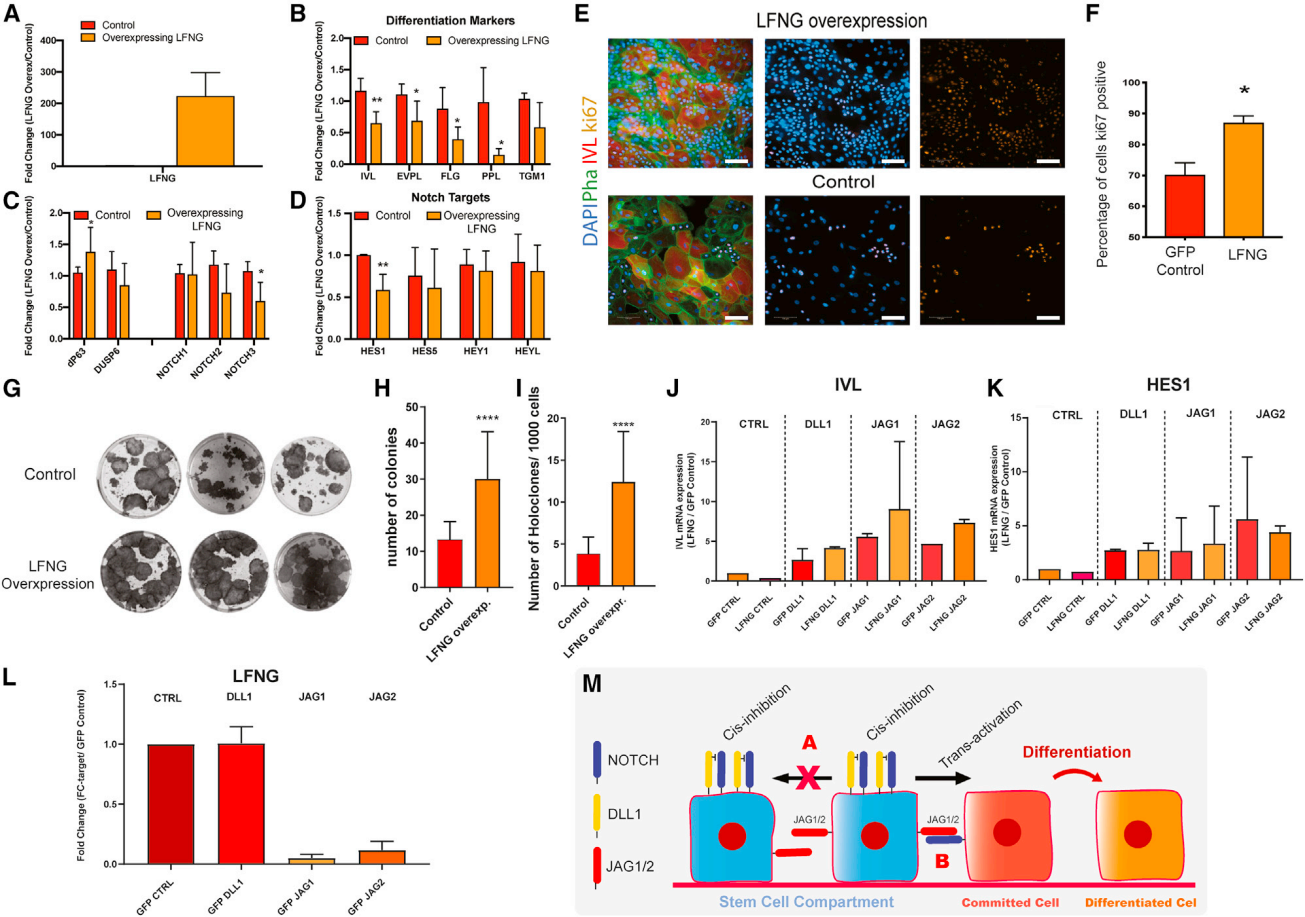
Effects of LFNG overexpression in cultured human keratinocytes (A–D) Expression profiles of (A) *LFNG*; (B) differentiation markers; (C) Notch receptors, *ΔP63* and *DUSP6*; and (D) Notch targets. Average fold change in mRNA abundance (normalized to expression of *RPS18*, *GAPDH*, and *TBP*) compared to control condition. Error bars represent standard deviation. One-way ANOVA with Holm Sidak’s multiple comparisons test. ^∗^p < 0.05, ^∗∗^p < 0.01, ^∗∗∗^p < 0.001. N = 3 independent samples. (E and F) Ki67-positive cells determined by immunostaining. (E) Representative confocal images. Scale bar represents 100 μm. (F) Percent Ki67-positive IVL-negative cells. Two-tailed, unpaired Student’s t test. ^∗^p < 0.05. N = 3 independent samples. (G–I) Effect of LFNG overexpression on colony formation. Three technical replicates from three different lentiviral infections. (G) Representative wells stained with Rhodanile blue. (H) Total colonies per well. (I) Stem cell colonies per well, defined as large colonies. (J–L) Effect of LFNG or GFP (control) overexpression on response to exogenous DLL1, JAG1, JAG2, or anti-B2MG (control). Standard errors are shown. N = 3 (J and K) and N = 2 (L) independent samples. (M) Schematic. Epidermal stem cells express LFNG, Jagged1/2, and DLL1. LFNG induces *cis* inhibition based on Notch-DLL1 interaction (A) leaving available Jagged ligands in the membrane. Jagged ligands on the surface of stem cells can only activate cells that do not have LFNG activity (B). The receiving cell activates Notch signaling and thereby initiates terminal differentiation. See also Figure S4.

To test whether LFNG overexpression in cultured keratinocytes would expand the stem cell compartment, we overexpressed LFNG using a lentiviral vector. As controls, we overexpressed GFP (Figure 4A) or transduced cells with the empty LFNG vector (Figure S4A). Expression of terminal differentiation markers and HES1 was reduced in cells overexpressing LFNG (Figures 4B, 4D, and S4B). Conversely, there was an increase in ΔP63 (Figure 4C).

We detected more Ki67-positive cells in cultures overexpressing LFNG when compared with the controls (Figures 4E and 4F). There was a significant increase in the total number of colonies and the number of large clones formed by keratinocytes overexpressing LFNG (Figures 4G–4I, S4C, and S4D). LFNG overexpression did not affect keratinocyte responsiveness to Notch ligands (Figures 4J and 4K). Nevertheless, there was a reduction in *LFNG* expression in cells exposed to Jagged1 and Jagged2 but not to DLL1 (Figure 4L).

These results suggest that LFNG expression amplifies the effects of DLL1 in epidermal stem cells (Figure 4M).

## Discussion

By reanalyzing published scRNA-seq data, we have been able to test whether stem cell characteristics defined in culture reflect the *in vivo* situation. The data analysis led us to predict that LFNG amplifies the effects of DLL1 in stem cells and thereby design mechanistic experiments.

The clustering data validated the conclusion from earlier experimental studies that there are subpopulations of basal cells that differ in their capacity for self-renewal (Tan et al., 2013). PAGA analysis indicates that *Basal II* cells are the most stem-like state. The scRNA-seq data also allowed identification of new stem cell markers, such as *COL17A1*. Col17A1 protein expression is enriched in basal keratinocytes that lie where the epidermis projects into the underlying dermis (Wang et al., 2020). This is the location of cells with high expression of the stem cell markers *ITGB1, CSPG4*, and *CD46* (based on flow cytometry and immunofluorescence microscopy) and *DLL1* (based on *in situ* hybridization) (Lowell et al., 2000; Tan et al., 2013). One unexpected feature of *Basal I*, *II*, and *III* is that markers that are co-expressed by protein detection methods are differentially expressed at the transcript level. This may reflect differences in protein turnover rates. For example, the turnover rates calculated from cultured breast epithelial cells are approximately 15 h for ITGB1, 23 h for ITGA6, and 45 h for CAV1 (Ly et al., 2018).

In culture, transient upregulation of an interacting network of protein phosphatases acts as an unstable commitment switch between the stem cell and differentiated cell states (Mishra et al., 2017). Unbiased analysis of human epidermis identified two transitional cell populations in which *DUSP6* and *DUSP10* were upregulated. Notch signaling activation was primarily associated with *Transition I* and *II,* on the basis of *HES1*, *IRF6*, *FBXW7*, *NEDD4L*, *DTX2*, and *SIRT1* expression (Estrach et al., 2006; Nguyen et al., 2006). The spatial distribution patterns of Notch ligands and receptors in mouse and human skin (reviewed by Watt et al., 2008) are consistent with the scRNA-seq data.

DLL1 expression protects stem cells from differentiation, mediates stem cell clustering, and instructs neighboring cells to differentiate (Lowell et al., 2000; Negri et al., 2019). However, it is puzzling that DLL1 exerts these effects while being expressed at much lower levels than JAG1 and JAG2, which promote differentiation. scRNA-seq revealed that *LFNG*, like *DLL1*, is most highly expressed in *Basal II*. LFNG increases Notch affinity for DLL1 and decreases affinity for Jagged (Luca et al., 2017; Kakuda and Haltiwanger, 2017). Overexpression of LFNG in cultured human keratinocytes decreased differentiation and promoted colony formation.

Our results suggest that LFNG amplifies the effects of DLL1 in protecting stem cells from undergoing differentiation (Figure 4M). LFNG could act by inducing *cis* inhibition based on Notch-DLL1 interaction, thereby protecting cells from JAG2 signals. *Cis* inhibition between Notch and DLL1 would increase Jagged availability in the plasma membrane (Figure 4M), allowing cells to simultaneously display *cis* inhibition and a sending-signal state based on Jagged. In this way, *Basal II* stem cells could be protected from differentiation and at the same time stimulate neighboring cells to differentiate.

It will be of interest to develop techniques to isolate keratinocyte populations corresponding to *Basal I, II*, and *III*, in order to examine post-translational modification of Notch proteins. Furthermore, by FISH, *LFNG* expression appeared to be uniform in the basal layer, raising the question of to what extent the *Basal II* state is spatially patterned. Additional analysis of the effects of knocking down *LFNG* in keratinocytes and of knockdown and overexpression in skin reconstitution assays will provide more information about the role of Notch signaling in epidermal homeostasis. Extending our bioinformatic analysis to include immune cells and melanocytes may provide new insights into the role of Notch and other signaling pathways in mediating keratinocyte interactions with other cell types.

## Experimental procedures

### Resource availability

#### Corresponding author

Further information and requests for resources and reagents should be directed to and will be fulfilled by the corresponding author, Fiona Watt (fiona.watt@kcl.ac.uk).

#### Materials availability

All unique/stable reagents generated in this study are available from the corresponding author on completion of a Materials Transfer Agreement.

### scRNA-seq

Quality control metrics for the scRNA-seq data were described previously (Reynolds et al., 2021). Data analysis, dimensionality reduction, cell clustering, and gene profile expression were performed using Seurat’s package (v.3.1.5) in R programming language and R-studio (version 1.2.5033) (see supplemental information).

### Keratinocyte culture

Neonatal foreskin normal human keratinocytes (strains km and kn) were cultured, as described previously, on a mitotically inactivated feeder layer of J2-3T3 cells in FAD medium (Tan et al., 2013). See supplemental information for differentiation, clonogenicity, and proliferation assays.

### RNA extraction and real-time quantitative PCR

Cells were lysed in RLT Lysis Buffer (Qiagen) containing 1% β-mercaptoethanol (Sigma-Aldrich). RNA was isolated using the Qiagen RNeasy mini kit (Qiagen). Complementary DNA synthesis was performed using the SuperScript III Reverse Transcriptase kit (Life Technologies). Real-time quantitative PCR reactions were implemented using TaqManTM probes (Invitrogen) or specifically designed primers (Tables S1 and S2). See supplemental information for real-time quantitative PCR reactions and RNA isolation from tissue.

### Functionalized substrates

Six-well plates (Falcon) were coated with human fibronectin (4 μg/cm^2^) and recombinant protein G (Sigma-Aldrich) in PBS and then incubated with Fc-tagged ligand and control recombinant proteins (2.5 μg/cm^2^) (Negri et al., 2019), as described in supplemental information.

### Lentivirus transduction

10^6^ keratinocytes were seeded per well in collagen type-1 coated six-well plates containing KSFM. After 24 h, 100 μL of lentiviral particles and 5 μg/ml polybrene (Sigma-Aldrich) were added. The following day, the medium was replaced with KSFM containing puromycin (2 μg/ml, Thermo Fisher Scientific). After 48 h, cells were harvested and replated in FAD on feeder cells. The lentivirus constructs were MISSION LentiORF LFNG, MISSION TRC3 ORF GFP Lentivirus Control (Sigma-Aldrich), and pLX307, the commercially available version of pLX_TRC317.

### Immunostaining and image analysis

Frozen sections of adult human breast skin were fixed with 4% paraformaldehyde for 20 min and stored at −80°C prior to staining. Sections were blocked and incubated overnight with primary antibodies: anti-keratin14 (Biolegend, 906001) and anti-CD74 (Abcam, ab64772). Slides were washed three times with PBS and labeled with DAPI and secondary antibodies for 1 h (A21206, A32570, A2287, Thermo Fisher Scientific). Samples were mounted in ProLong 394 Gold anti-fade (Thermo Fisher Scientific).

### RNAscope

Human skin samples (obtained with informed consent, subject to both institutional and external research ethics council (REC) review (REC reference 19/NE/0063) were embedded in Tissue-Tek O.C.T. (Life Technologies, Waltham, MA, USA) and stored at −80°C. 10-μm sections were cut with a Thermo Cryostar Nx70 (Thermo Fisher Scientific, Waltham, MA, USA) and placed on SuperFrost Plus glass slides (Thermo Fisher Scientific, ref J2800AMN2). Sections were labeled using the RNAscope Multiplex Fluorescent Detection Kit v2 (ACDBio, Newark, California, USA, cat. no. 323100). See supplemental information for more information.

### Statistical tests and graphing

Statistical analysis was performed using GraphPad Prism (version 7.0). Data are shown as the mean and standard error of the mean unless otherwise stated. Data were analyzed using Student’s t tests, one or two-way ANOVA with Tukey’s multiple comparison post test. p < 0.05 was considered significant. Graphs were obtained using GraphPad Prism 7 or ggplot2 R package version 3.1.0 (https://ggplot2.tidyverse.org).

## Data Availability

The scRNA-seq dataset used in this study has been deposited in ArrayExpress: www.ebi.ac.uk/arrayexpress/experiments/E-MTAB-8142.

## Supplemental information

Document S1. Supplemental experimental procedures, Figures S1–S4, and Tables S1 and S2

Document S2. Article plus supplemental information
