# Supplementary material for: Single-cell RNA sequencing of human epidermis identifies Lunatic fringe as a novel regulator of the stem cell compartment
Source: Stem Cell Reports. 2023 Oct 12;18(11):2047–55. doi: 10.1016/j.stemcr.2023.09.007 (PMC10679657; doi:10.1016/j.stemcr.2023.09.007)
Supplement: Document S1. Supplemental experimental procedures, Figures S1–S4, and Tables S1 and S2 [file mmc1.pdf]

Stem Cell Reports, Volume *18*

Supplemental Information

Single-cell RNA sequencing of human epidermis identifies Lunatic fringe as a novel regulator of the stem cell compartment

Victor Augusti Negri, Blaise Louis, Sebastiaan Zijl, Clarisse Ganier, Christina Philippeos, Shahnawaz Ali, Gary Reynolds, Muzlifah Haniffa, and Fiona M. Watt

Supplementary Material

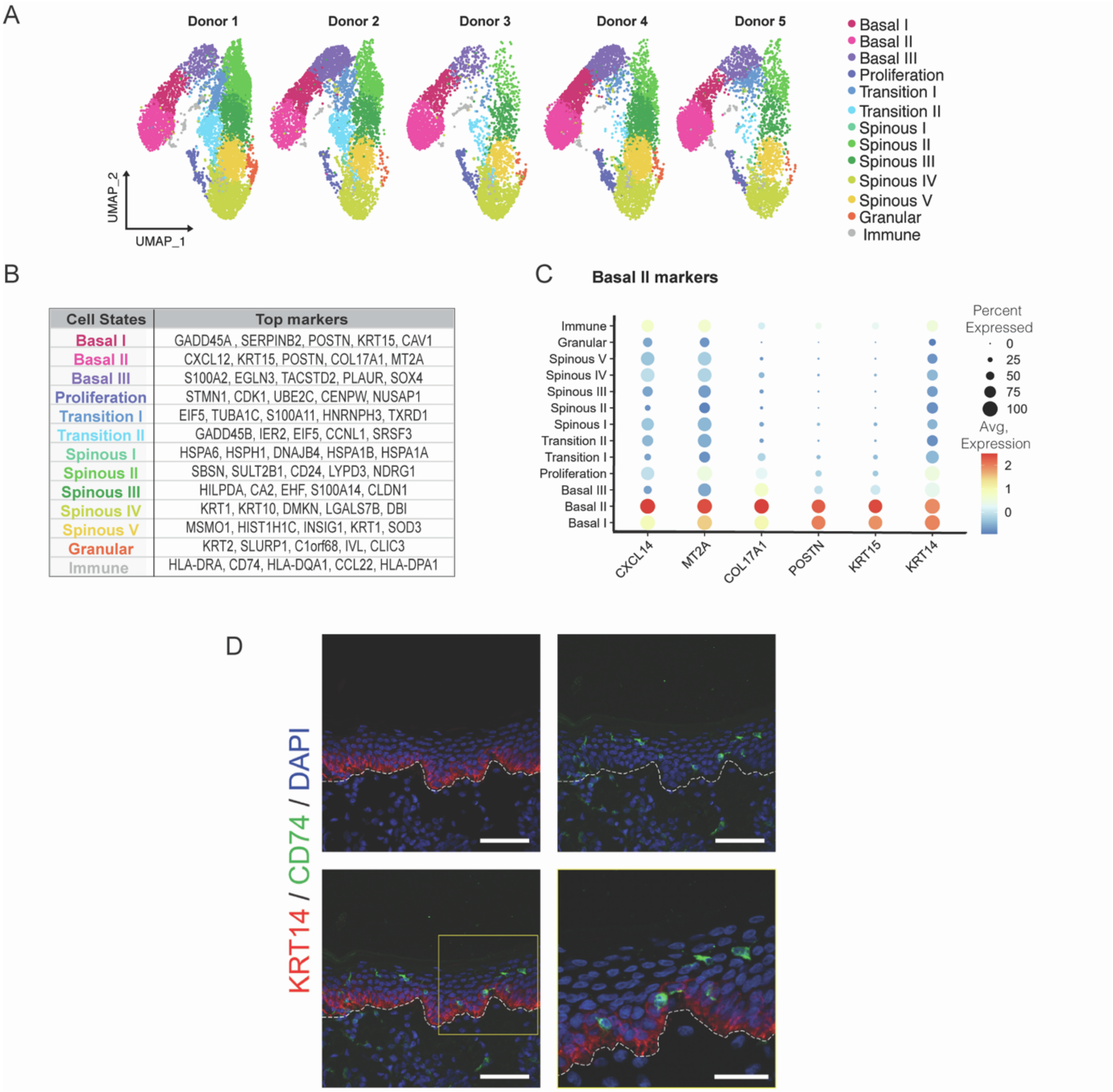

Cell States	Top markers
Basal I	GADD45A , SERPINB2, POSTN, KRT15, CAV1
Basal II	CXCL12, KRT15, POSTN, COL17A1, MT2A
Basal III	S100A2, EGLN3, TACSTD2, PLAUR, SOX4
Proliferation	STMN1, CDK1, UBE2C, CENPW, NUSAP1
Transition I	EIF5, TUBA1C, S100A11, HNRNPH3, TXRD1
Transition II	GADD45B, IER2, EIF5, CCNL1, SRSF3
Spinous I	HSPA6, HSPH1, DNAJB4, HSPA1B, HSPA1A
Spinous II	SBSN, SULT2B1, CD24, LYPD3, NDRG1
Spinous III	HILPDA, CA2, EHF, S100A14, CLDN1
Spinous IV	KRT1, KRT10, DMKN, LGALS7B, DBI
Spinous V	MSMO1, HIST1H1C, INSIG1, KRT1, SOD3
Granular	KRT2, SLURP1, C1orf68, IVL, CLIC3
Immune	HLA-DRA, CD74, HLA-DQA1, CCL22, HLA-DPA1

Figure S1 related to Figure 1. A*:* UMAP plot showing the 13 distinct cell states found in the 5 distinct donors used for this study B*:* Top 5 genes differentially expressed in each cell state. For this analysis we applied Wilcoxon rank sum test and considered genes as differentially expressed when the average log fold change test (avg_logFC) was higher than 0.5 and p-value for multiple testing (Bonferroni) was lower than 0.001. C: DotPlot showing scaled normalized expression of selected genes differentially expressed in *Basal II* cluster*.* D: Adult human skin labelled with antibodies to CD74 (green) and Krt14 (red) with DAPI nuclear counterstain (blue). Scale bars: 60µm, 30µm (high magnification of boxed area).

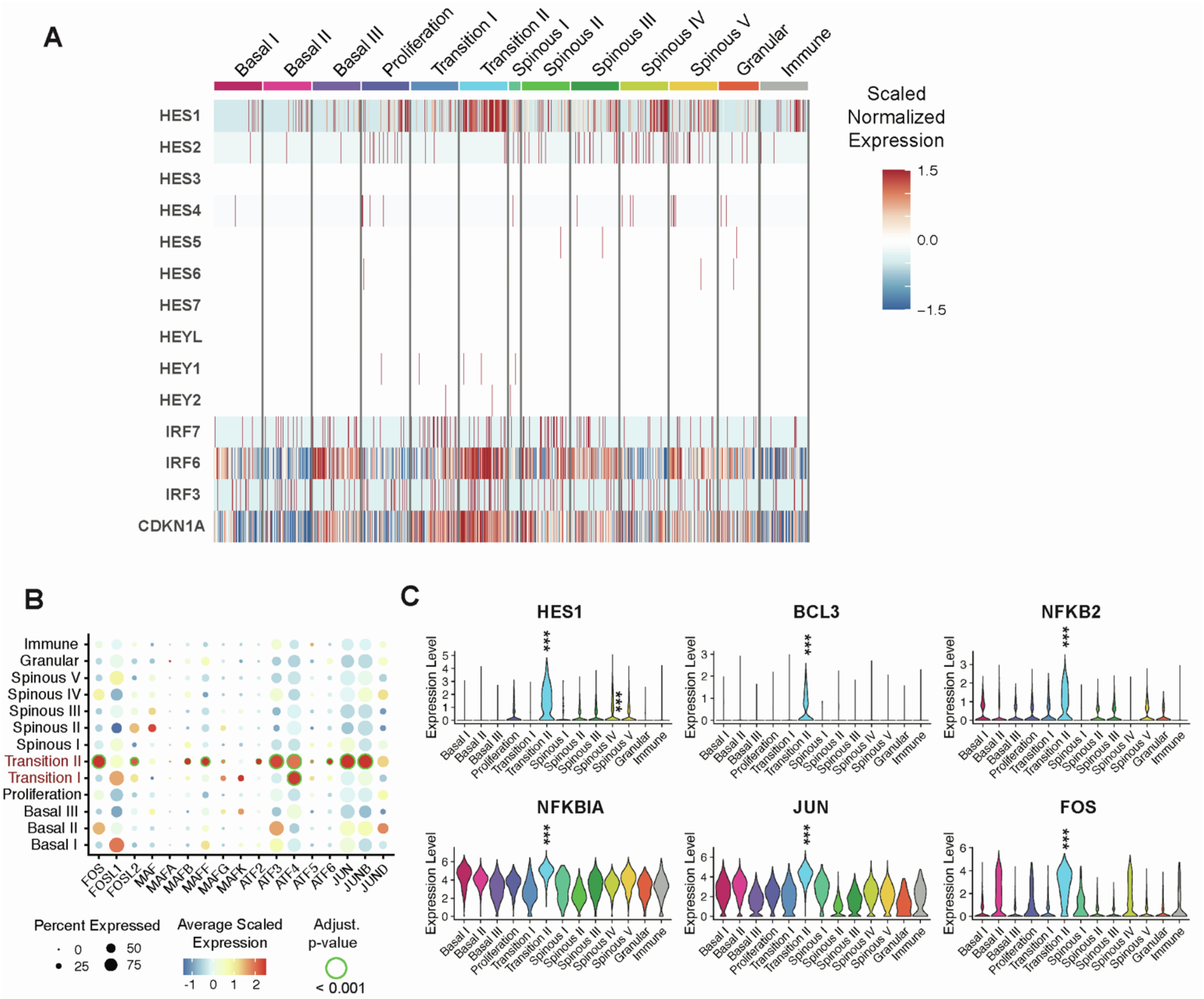

Figure S2 related to Figure 2. A: Heatmap plot showing scaled expression values of known Notch pathway target genes in epidermal cells (n = 5, 27138 cells). B: Dot plot showing expression distribution of AP1 factors in distinct epidermal cell states. Green circles: adjusted p-value < 0.001 (Bonferroni Correction). C: Violin plots showing normalized expression distribution of a selection of markers in the distinct cell states. Adjusted p-value <0.001 (***).

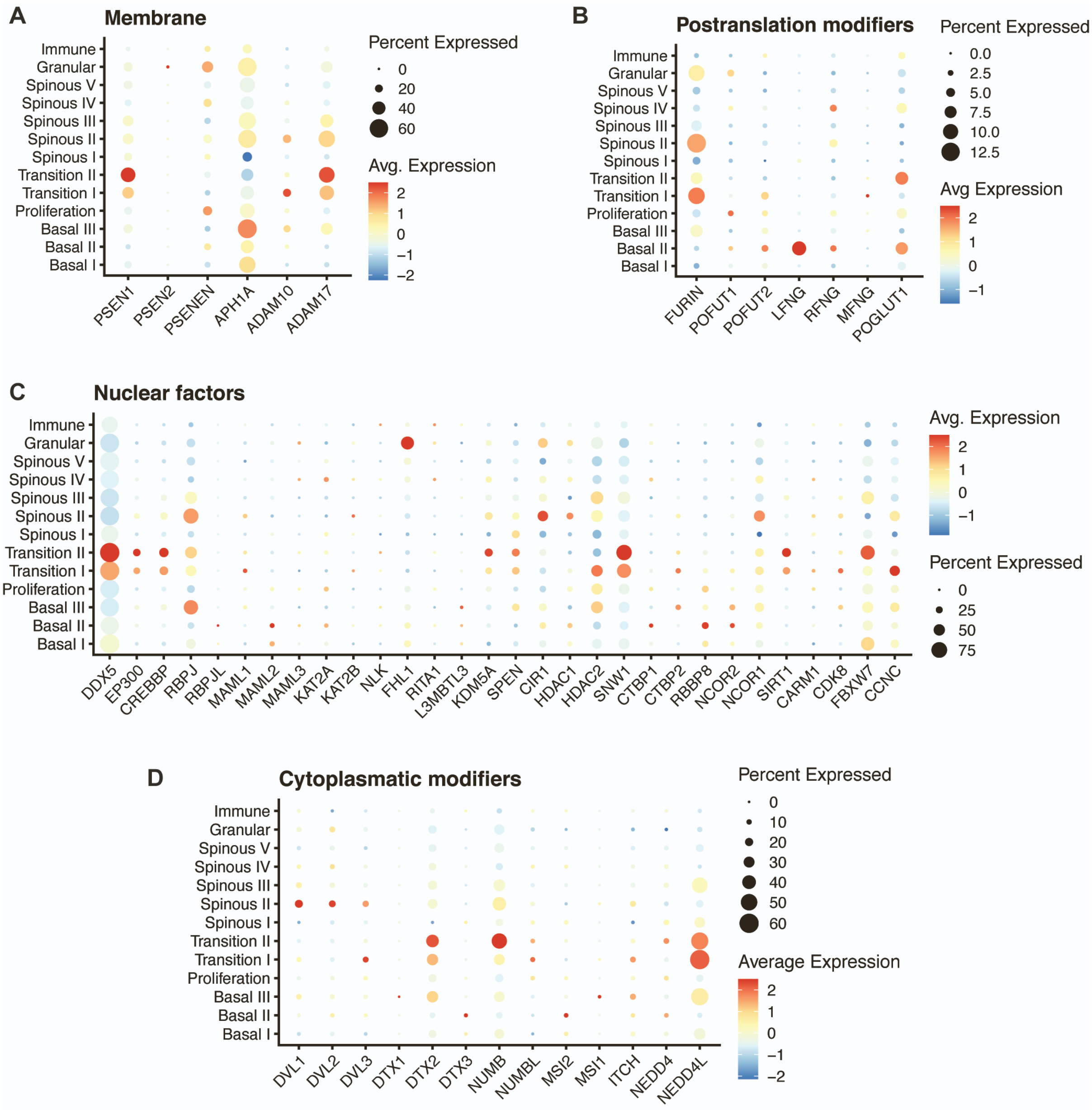

Figure S3 related to Figure 2. Dot plots showing distribution of the normalized expression of Notch signalling pathway regulators. A: Notch associated genes encoding proteins present in the plasma membrane; B: genes associated with Notch receptor and ligand posttranslational modifications; C: nuclear Notch regulatory factors; D: Notch cytoplasmic regulatory factors.

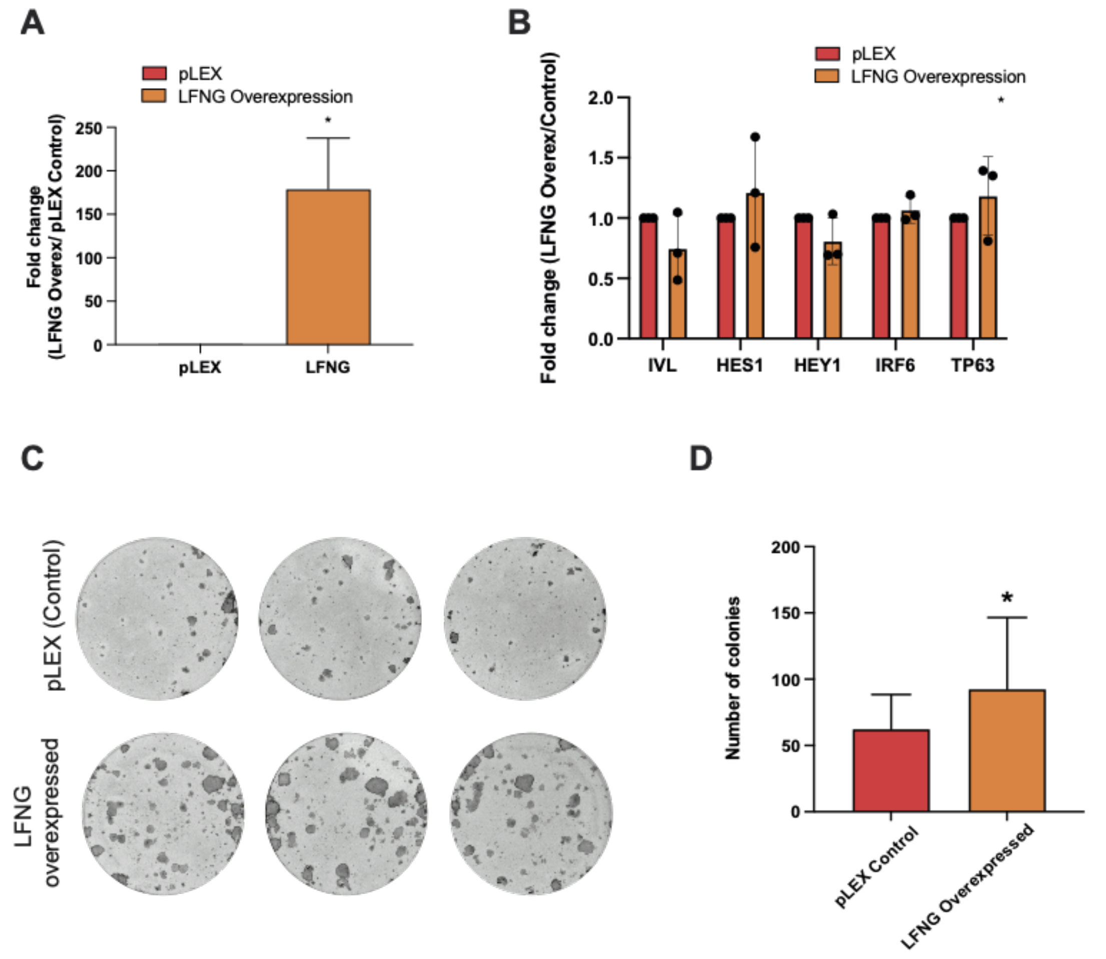

Figure S4 related to Figure 4. LFNG overexpression in primary human keratinocytes. A: Expression levels by qPCR of LFNG in LFNG overexpressed keratinocytes and pLEX controls. B: Expression levels of Notch targets and terminal differentiation markers in LFNG overexpressing keratinocytes and control (pLEX). C-D: Effect of LFNG overexpression on colony formation. 1000 cells were seeded per well. Assays were performed on three technical replicates from three different lentiviral infections. C: Representative wells stained with 1% Rhodanile Blue. D: Total number of colonies per well. P value of 0.05 or less was considered statistically significant (*). N=3.

Supplemental Table 1 - TaqMan probes used for the qPCR reactions (related to Experimental Procedures)

Gene Name	Gene Symbol	TaqMan Assay ID
Delta-Like Ligand 1	DLL1	Hs.379912_m1
Glyceraldehyde-3-phosphate dehydrogenase	GAPDH	Hs02758991_g1
Hes family bHLH transcription factor 1	HES1	Hs00172878_m1
Hes family bHLH transcription factor 2	HES2	Hs01021800_g1
Hes family bHLH transcription factor 4	HES4	Hs00970270_g1
Hes family bHLH transcription factor 5	HES5	Hs01387463_g1
Hes family bHLH transcription factor 6	HES6	Hs05043218_s1
Hes family bHLH transcription factor 7	HES7	Hs00261517_m1
Hes related family bHLH transcription factor with YRPW motif 1	HEY1	Hs01114113_m1
Interferon regulatory factor 6	IRF6	Hs01062178_m1
Involucrin	IVL	Hs00846307_s1
Jagged 1	JAG1	Hs01070032_m1
Jagged 2	JAG2	Hs00171432_m1
Lunatic Fringe	LFNG	Hs00385436_g1
Manic Fringe	MFNG	Hs00159117_m1
Radical Fringe	RFNG	Hs01357010_g1
Ribossomal protein S18	RPS18	Hs01375212_g1
TATA-box binding protein	TBP	Hs00427620_m1
Tumor protein p63	TP63	Hs00978340_m1
Hes related family bHLH transcription factor with YRPW motif 2	HEY2	Hs01012057_m1
Hes related family bHLH transcription factor with YRPW motif-like	HEYL	Hs01113778_m1

Supplemental Table 2 - Primer pairs used in SYBR green RT qPCR reactions (related to Experimental Procedures)

Gene Name	Gene Symbol	Primer Forward	Primer Reverse
Hes family bHLH transcription factor 1	HES1	AAAAATTCCTCGTCCCCGGT	ATGCCGCGAGCTATCTTTCT
Ribossomal protein S18	18sRNA	GCAATTATTCCCCATGAACG	GGCCTCACTAAACCATCCAA
DeltaNp63 - p63 isoform	dNtp63	TCCTCAGGGAGCTGTTATCC	TGACTAGGAGGGGCAATCTG
Dual Specificity Phosphatase 6	DUSP6	ACCTGGAAGGTGGCTTCAGT	CTCGGTCAAGGTCAGACTCG
Hes related family bHLH transc. factor with YRPW motif1	HEY1	GTTCGGCTCTAGGTTCCATGT	CGTCGGCGCTTCTCAATTATTC
Interferon regulatory factor 6	IRF6	GCTCTCTCCCAATGACCTGGA	CCATGACGTCCAGCAGCTTGCTA
Integrin subunit alfa 6	ITGA6	CGCTGGGATCTTGATGCTTGCT	TGAGCATGGATCTCAGCCTTGTGA
Integrin subunit beta 1	ITGB1	GACGCCGCGCGGAAAAGATG	ACCACCCACAATTTGGCCCTGC
Involucrin	IVL	TCCTCCAGTCAATACCCATC	CAGCAGTCATGTGCTTTTCCT
Notch Receptor 1	NOTCH1	TCCACCAGTTTGAATGGTCA	AGCTCATCATCTGGGACAGG
Notch Receptor 2	NOTCH2	GATCACCCGAATGGCTATGAAT	GGGGTCACAGTTGTCAATGTT
Notch Receptor 3	NOTCH3	TGGCGACCTCACTTACGACT	CACTGGCAGTTATAGGTGTTGAC
Notch Receptor 4	NOTCH4	TGTGAACGTGATGTCAACGAG	ACAGTCTGGGCCTATGAAACC
NUMB, endocytic adaptor protein	NUMB	GGCATACAGAGGTTCCTACA	TGCTCCTTTGACCGCTAC
Periplakin	PPL	GCAGAGTGACCTGGCTCGGCT	GCCGCATCCGCCTCTAGCAC
TATA-box binding protein	TBP	GTGACCCAGCATCACTGTTTC	GAGCATCTCCAGCACACTCT
Transglutaminase 1	TGM1	GCACCACACAGACGAGTATGA	GGTGATGCGATCAGAGGAT

Supplemental Experimental Procedures (related to Experimental Procedures)

scRNAseq analysis

To reconstruct the keratinocyte differentiation programme we converted the Seurat object in a SCANPY H5AD file using the SeuratDisk package (Stuart et al., 2019; Hoffman, 2021) and applied Partition-based approximate graph abstraction (PAGA) using Scanpy v1.9.1 (sc.tl.paga) on python version 3.9.5 (Wolf et al., 2018).

Raw counts were normalized using NormalizedData (scalefactor = 1000, method = LogNormalize) and scaled using ScaleData. Principal component analysis (PCA) was performed using Seurat's RunPCA function and considering the 2000 most variable genes (obtained with FindVariableFeatures Seurat's function). Uniform manifold approximation and projection (UMAP) dimensional reduction was obtained based on the 30 first principal components (Pei et al., 2018). Next, we determined the k-nearest neighbor graph using the FindNeighbors function. Cell clustering was performed by Louvain's clustering using FindClusters function (resolution = 0.5). To label and identify the clusters, markers were obtained with FindAllMarkers applying the non-parametric Wilcoxon Rank Sum test. Distinct cell states were annotated manually based on known markers and the most significantly expressed genes in each cluster (adjusted p-value adjusted < 0.001, Bonferroni correction) (Figure S1). Application of DoubletFinder (McGinnis et al., 2019) ruled out the possibility that the Immune cell state consisted of cell doublets. Differential gene expression was determined using FindMarkers and the non-parametric Wilcoxon Rank Sum test. Genes with a log-fold change > 0.5 and adjusted P value (Bonferroni correction) < 0.001 were considered differentially expressed.

Keratinocyte culture

Complete FAD medium comprises 1-part Ham's F12 medium, three parts DMEM and $1.8x10^{-4}$ M adenine (Life Technologies), 10 % foetal bovine serum (Gibco), 0.5 µg/ml, hydrocortisone (Thermo Fisher Scientific), 5 µg/ml insulin (Sigma Aldrich), 10^{-10}M cholera toxin (Enzo-Life Science), 100 µg/ml streptomycin (Life Technologies), 100U/ml penicillin (Life Technologies), 10 ng/ml epidermal growth factor (EGF), 450 µg/ml glutamine (Life Technologies) n some experiments keratinocytes were transferred, feeder-free, to collagen type-1 (20µg/mL) coated surfaces in keratinocyte serum-free medium (KSFM, Thermo Fisher Scientific) supplemented with bovine pituitary extract (30µg/ml) and epidermal growth factor (EGF, 0.2 ng/ml, Thermo Fisher Scientific) (complete KSFM). To stimulate

differentiation prior to RNA isolation $1x10^5$ cells per plated per well of 12 well-plates in complete KSFM medium for 24 hours and then transferred to FAD medium for 48 or 72 hours.

For clonogenicity assays 10^3 cells were plated per well of 6-well plates containing J2-3T3 feeders and then fixed and stained with Rhodanile Blue as described previously (Mishra et al., 2017). Colony formation was quantitated using a Molecular Imager Gel Doc XR+ (Bio-Rad) and ImageJ software.

For proliferation evaluation cells were seeded for 48 hours in FAD in a 96 well plate and labelled with anti-Ki67, DAPI (Invitrogen) and phalloidin (Alexa FluorTM 647 Phalloidin, Thermo Fisher Scientific). Plates were imaged using an Operetta (Perkin-Elmer), and images were quantified using the Harmony® high-content analysis software package (Perkin- Elmer). In the analysis pipeline (Louis et al., 2022) nuclei were identified by DAPI staining and incomplete cells at the borders of the images were excluded. A staining intensity threshold was used to identify Ki67 positive nuclear staining relative to background.

Real-Time Quantitative PCR reactions and RNA isolation from tissue
RT-qPCR reactions were performed with the SYBRT^M Green PCR Master Mix (Thermo Fisher Scientific) or on a CFX 384 Touch RT- qPCR machine (Bio-Rad). Ct values were normalized to the ct values of 18S, GAPDH and TBP (housekeeping genes). Expression values were obtained applying the -ΔΔCT method (Livak and Schmittgen, 2001).

To isolate RNA from human skin (surgical waste, obtained under NHS Research Ethics approval 19/NE/0063), the tissue was minced with a scalpel and incubated for 12 hours at 4^oC in 0.2% Dispase (Corning) prior to separating the epidermis from the dermis with tweezers. The skin fragments were transferred to tubes containing TrypLE (Thermo Fisher Scientific) for 10 minutes. Undigested material was discarded following centrifugation at 150 x g for 5 minutes. After two washes with PBS, total RNA was extracted using the PureLink-RNA Mini-Kit (Thermo Fisher Scientific).

Functionalised substrates
Human recombinant proteins were as follows: Jagged 2 Fc Chimera, CF (RnD systems), Jagged 1 Fc Chimera (RnD systems), DLL1 Fc chimera (Adipogen). Beta-2-microglobulin (clone B2M-01, IgG2a) antibody was obtained from Abcam. Human keratinocytes were trypsinized with the addition of DAPT (10µM final, gama-secretase inhibitor, Sigma-Aldrich). Cells ($5x10^4$) were added to the plates containing KSFM medium supplemented with DAPT (10µM, Sigma Aldrich). After 3 hours, the medium was removed, and KSFM medium supplemented with calcium ($CaCl_2$, 1.2mM) was added.

RNAscope
RNA integrity was confirmed with housekeeping control probes (high (UBC), medium (PPIB) and low (POLR2A) expression). A 20zz probe of human LFNG mRNA was designed by the ACD Probe Design Team targeting 296-1480 of NM_001040167.2 of the LFNG Sequence in FASTA format. This design targets all 4 transcript variants on NCBI. The Opal 520 Reagent Pack (Akoya Biosciences, Marlborough, Massachusetts, USA, ref : FP1487001KT) was used at a dilution of 1:1,000 for the fluorophore step to develop the channel associated with the LFNG probe. Labelling was followed by three 5 minute washes in PBS containing Tween (0.1%), followed by incubation in blocking buffer containing 10% serum, 0.2% fish skin gelatin, 0.1% BSA, and 0.5% Tween-20 (all Sigma-Aldrich, St. Louis, MO, USA) in PBS. Sections were labelled with Chicken IgY anti-Human Keratin 14 primary antibody (Biolegend, Inc, San Diego, CA, USA, ref 906001) in blocking buffer overnight at 4°C. Sections were washed with PBS and then labelled with Goat anti-Chicken IgY (H+L) Secondary Antibody conjugated with Alexa Fluor™ 647 (Thermo Fisher Scientific, ref A21449) for 1 hour at room temperature, followed by PBS washes. Nuclei were counterstained with 4′,6-diamidino-2-phenylindole and mounted using ProLong Gold Antifade Mountant (Thermo Fisher, cat. no. P36930). Slides were imaged with a Nikon A1 upright confocal microscope (Nikon, Tokyo, Japan) using a 20 dry lens and were processed using Fiji software.

Supplemental Acknowledgements (related to Acknowledgements)
This work was supported by grants to FMW from the UK Medical Research Council (MR/PO18823/1) and the Wellcome Trust (206439/Z/17/Z). VAN is the recipient of a National Council for Scientific and Technological Development-Brazil (CNPq) doctoral scholarship. BL and SZ received support from the Danish National Research Foundation (DNRF135). MH is funded by Wellcome (WT107931/Z/15/Z), The Lister Institute for Preventive Medicine and NIHR and Newcastle-Biomedical Research Centre. MH and FMW acknowledge funding from the Wellcome Human Cell Atlas Strategic Science Support (WT211276/Z/18/Z). FMW also acknowledges funding from the Department of Health via the National Institute for Health Research comprehensive Biomedical Research Centre award to Guy's & St Thomas' National Health Service Foundation Trust in partnership with King's College London and King's College Hospital NHS Foundation Trust.

Supplemental References (related to Supplemental Experimental Procedures)

Hoffman, P. (2021) SeuratDisk: interfaces for HDF5-based single cell file formats.

Livak, K. J. and Schmittgen, T. D. (2001) Analysis of relative gene expression data using real-

Louis, B., Tewary, M., Bremer, A.W., Philippeos, C., Negri, V.A., Zijl, S., Gartner, Z.J., Schaffer, D.V., and Watt, F.M. (2022) A reductionist approach to determine the effect of cell-cell contact on human epidermal stem cell differentiation. Acta Biomater. *150*, 265-276.

McGinnis, C.S., Murrow, L.M., and Gartner, Z.J. (2019). DoubletFinder: doublet detection in single-cell RNA sequencing data using artificial nearest neighbors. Cell Syst. *8*, 329-337.

Stuart, T., Butler, A., Hoffman, P., Hafemeister, C., Papalexi, E., Mauck, W.M. 3rd, Hao, Y., Stoeckius, M., Smibert, P., and Satija, R. (2019) Comprehensive integration of single-cell data. Cell *177*, 1888-1902.

Stuart, T., Butler, A., Hoffman, P., Hafemeister, C., Papalexi, E., Mauck, W.M. 3rd, Hao, Y., Stoeckius, M., Smibert, P., and Satija, R. (2019) Comprehensive integration of single-cell data. Cell *177*, 1888-1902.

Wolf, F. A., Angerer P., Theis, F.G. (2018) SCANPY: Large-scale single-cell gene expression data analysis. Genome Biol. *19,* 15.
