# Supplementary material for: Single-cell RNA sequencing of human epidermis identifies Lunatic fringe as a novel regulator of the stem cell compartment
Source: Stem Cell Reports. 2023 Oct 12;18(11):2047–55. doi: 10.1016/j.stemcr.2023.09.007 (PMC10679657; doi:10.1016/j.stemcr.2023.09.007)
Supplement: Document S2. Article plus supplemental information [file mmc2.pdf]

Stem Cell Reports
Report
OPEN ACCESS

Single-cell RNA sequencing of human epidermis identifies Lunatic fringe as a novel regulator of the stem cell compartment

Victor Augusti Negri,[1,5] Blaise Louis,[1,6] Sebastiaan Zijl,[1] Clarisse Ganier,[1] Christina Philippeos,[1] Shahnawaz Ali,[1] Gary Reynolds,[2] Muzlifah Haniffa,[2,3] and Fiona M. Watt[1,4,*]
[1]King's College London Centre for Stem Cells and Regenerative Medicine, Guy's Hospital, London, UK
[2]Biosciences Institute, Newcastle University, Newcastle upon Tyne, UK
[3]Wellcome Sanger Institute, Wellcome Genome Campus, Cambridge, UK
[4]Directors' Research Unit, European Molecular Biology Laboratory, Heidelberg, Germany
[5]Present address: Translational Science and Experimental Medicine, Research and Early Development, Respiratory & Immunology, BioPharmaceuticals R&D, AstraZeneca, Cambridge, UK
[6]Present address: Semarion Ltd, Cambridge, UK
*Correspondence: fiona.watt@kcl.ac.uk
https://doi.org/10.1016/j.stemcr.2023.09.007

SUMMARY

Single-cell RNA sequencing (scRNA-seq) of human skin provides a tool for validating observations from *in vitro* experimental models. By analyzing a published dataset of healthy adult epidermis, we confirm that the basal epidermal layer is heterogeneous, and three subpopulations of non-dividing cells can be distinguished. We show that Delta-like ligand 1 (DLL1) is expressed in a subset of basal cells previously identified as stem cells in cultured human keratinocytes and map the distribution of other Notch ligands and receptors to specific epidermal cell compartments. Although DLL1 is expressed at low levels, it is expressed in the same cell state as the Notch regulator, Lunatic -fringe (LFNG, O-fucosylpeptide 3-beta-N-acetylglucosaminyltransferase). Overexpression of LFNG amplifies the effects of DLL1 in cultured keratinocytes, increasing proliferation and colony-forming ability. We conclude that using scRNA-seq resources from healthy human skin not only validates previous experimental data but allows formulation of testable new hypotheses.

INTRODUCTION

The outermost skin layer, the epidermis, comprises a stratified squamous epithelium, the interfollicular epidermis (IFE), and adnexal appendages (Watt, 2014). IFE proliferation takes place mainly in the basal layer, where the stem cells reside, and on initiation of terminal differentiation, cells move through the suprabasal layers, finally differentiating into corneocytes.

One of the pathways that controls the balance between IFE differentiation and proliferation is the Notch pathway. Notch signaling induces growth arrest and terminal differentiation of cultured keratinocytes (Lowell et al., 2000; Watt et al., 2008). Activation of Notch targets is observed in the suprabasal layers of mouse epidermis (Estrach et al., 2006; Nguyen et al., 2006) and in cultured keratinocytes. Notch activity occurs predominantly in cells committed to, or initiating, terminal differentiation (Rangarajan et al., 2001; Blanpain et al., 2006; Negri et al., 2019). Conditional ablation of *NOTCH1* in mice increases proliferation and decreases differentiation.

Three Notch ligands are found in human epidermis: *DLL1*, *JAG1*, and *JAG2* (Watt et al., 2008). *DLL1* is expressed in clusters of cells in the basal layer of human and mouse fetal epidermis (Rangarajan et al., 2001; Estrach et al., 2008). In cultured human keratinocytes, DLL1 plays a role in the stem cell compartment, inhibiting Notch signaling via *cis* inhibition (Lowell et al., 2000; Negri et al., 2019).

Notch signal modulation can occur at different levels, including glycosylation of ligands and receptors, ubiquitylation, endocytosis, and trafficking (Irvine, 2008; Stanley and Okajima, 2010). Fringe proteins are N-acetylglycosyltransferases (Irvine, 2008; LeBon et al., 2014) that transfer N-acetylglucosamine to O-fucose residues in Notch EGF repeats in the Golgi complex (Irvine, 2008). These enzymes can change the Notch extracellular domain (NECD) affinity for Notch ligands in the same cell or an adjacent cell. Lunatic fringe (LFNG) and Manic fringe (MFNG) increase Notch affinity for DLL1 and inhibit JAG receiving signals (LeBon et al., 2014). Radical fringe (RFNG) can increase the affinity for both ligands (Irvine, 2008).

In vitro studies of human keratinocytes have identified stem cell markers, using colony formation as a quantitative readout of stem cell number (Lowell et al., 2000; Tan et al., 2013). These studies point to the existence of multiple subpopulations of cells in the epidermal basal layer, a conclusion supported by scRNA-seq of keratinocytes isolated directly from healthy adult human skin (Cheng et al., 2018; Wang et al., 2020; Reynolds et al., 2021; Negri and Watt, 2022). In the present study, we have analyzed the scRNA-seq data in more depth, to gain insights into the role of DLL1 in the stem cell compartment and the different stages of differentiation at which the Notch pathway is active.

This is an open access article under the CC BY license (http://creativecommons.org/licenses/by/4.0/).

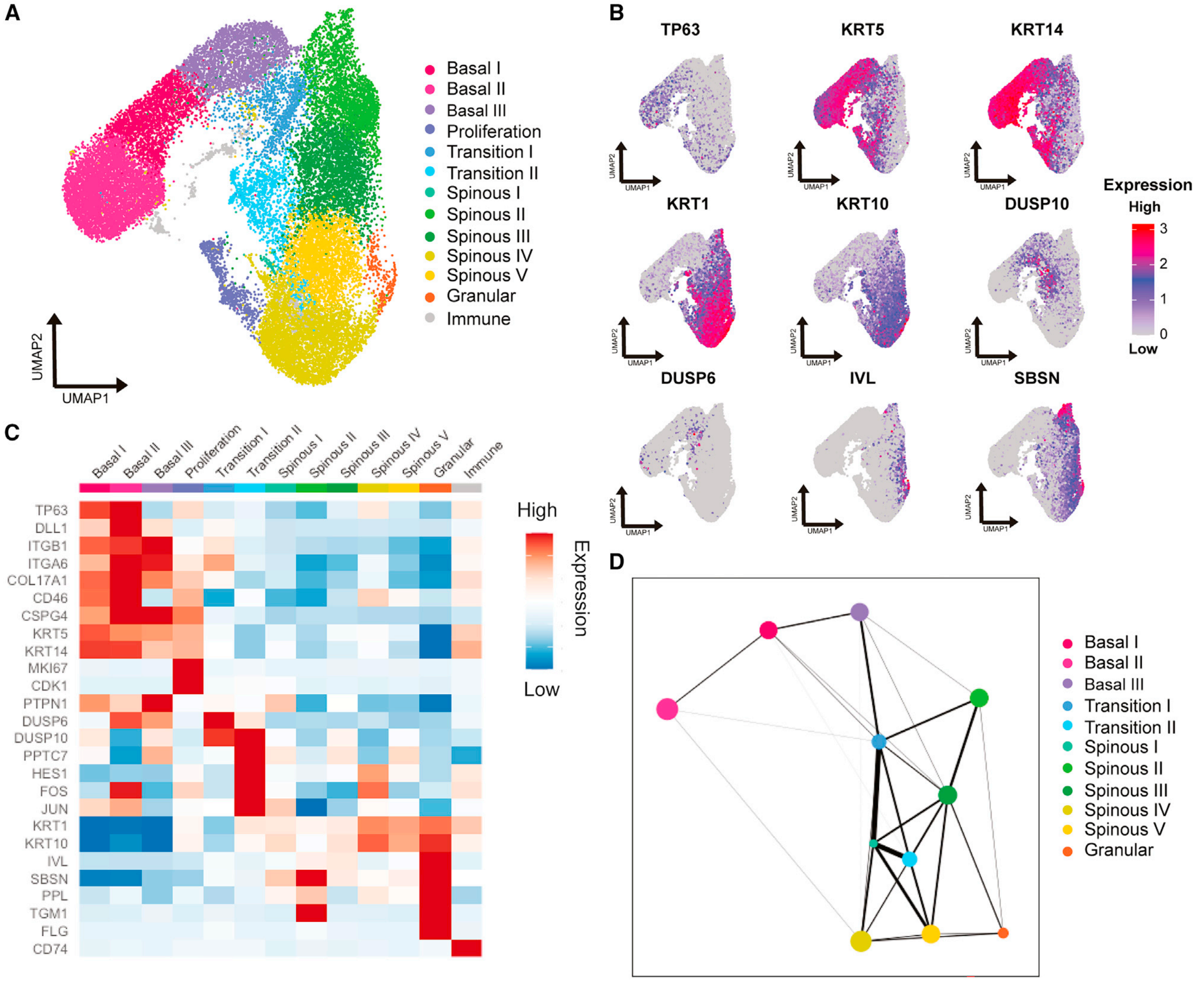

Figure 1. Transcriptional heterogeneity of human epidermal keratinocytes *in vivo*

(A and B) UMAP plots showing 13 distinct cell states found in human IFE (five donors, 27,138 cells) (A) and expression of known markers (B).

(C) Heatmap of averaged expression of genes differentially expressed in distinct cell states (log fold change).

(D) PAGA connectivity plot after removing the *Proliferation* and *Immune* states. Edge thickness corresponds to strength of connection between nodes. Node size reflects respective cluster (cell state) size.

See also Figure S1.

RESULTS

Mapping known epidermal markers to cell clusters identified by scRNA-seq

We explored Notch signaling using an unbiased approach with an scRNA-seq dataset of human IFE keratinocytes from five healthy adult donors (Reynolds et al., 2021; Negri and Watt, 2022). After quality control, clustering, and dimensionality reduction, we identified 13 distinct transcriptomic profiles (Figures 1A–1C and S1). All clusters were found in all five samples sequenced (Figure S1A). The five most highly expressed genes in each population are presented in Figure S1B.

Based on expression of basal layer markers such as *KRT5* and *14*, we assigned four clusters to the basal layer: *Basal I, II, III*, and *Proliferation*, the latter expressing high levels of cyclin-dependent kinase 1 (*CDK1*) and Marker of Proliferation Ki-67 (*MKI67*) (Figure 1C). The classic cell surface markers used to enrich for clonogenic human keratinocytes are integrins α2β1, α3β1, and α6β4. *ITGA3* and *ITGA6* were most abundant in *Basal II*, while *ITGA2* and *ITGB1* were most abundant in *Basal III*. *CD46*, identified

as a marker of Dll1+ stem cells (Tan et al., 2013), was most highly expressed in *Basal II*, as was *DLL1* (Figure 1C). *COL17A1*, *MT2A*, *CXCL24*, and *POSTN* were also expressed most highly in *Basal II* (Figure 1C). *CAV1* and *CAV2* are markers of *DLL1* high stem cells in culture (Tan et al., 2013), and *CAV1* was one of the most highly expressed cells in *Basal I* (Figure S1B).

We assigned two clusters as *Transition* based on expression of pro-commitment genes such as the protein phosphatases DUSP6 and DUSP10 (Mishra et al., 2017). Consistent with antibody labeling in human epidermis (Mishra et al., 2017), expression of *DUSP10* was more widespread than *DUSP6* (i.e., upregulated in *Transition I* and *II*) (Figure 1B).

Five clusters were ascribed to the first suprabasal (spinous) cell layers, based on expression of *KRT1* and *KRT10*. Granular cells were assigned based on *FLG* expression, the low number of cells in this cluster probably reflecting the difficulty in isolating cells from the uppermost IFE layers (Cheng et al., 2018).

We also identified a population of keratinocytes categorized as *Immune* based on co-expression of basal (*KRT5*, *KRT14*) and suprabasal (*KRT10*) markers and the macrophage migration inhibitory factor receptor *CD74*. A cluster of keratinocytes expressing *CD74* and other immune signature genes (Figure S1B) has been reported previously (Cheng et al., 2018). Immunostaining of human epidermis revealed scattered $CD74^{+}$ cells with a dendritic morphology in the basal and suprabasal cell layers (Figure S1). CD74 is expressed by dendritic cells (Su et al., 2017), and we speculate that the immune cluster in the scRNA-seq dataset comprises dendritic cells that have ingested keratinocyte RNA via trogocytosis (Zhao et al., 2022).

We used partition-based approximate graph abstraction (PAGA) to analyze connections between the different cell states (Figure 1D). We omitted *Immune* because it does not appear to be part of the normal differentiation trajectory and *Proliferation* because proliferation and differentiation are under separate control (e.g., Mishra et al., 2017). The strongest inferred trajectory was cells moving through *Basal II* to *Basal I* to *Basal III* and then into *Transition I*. This supports the definition of *Basal II* as the most stem-like state based on *DLL1* expression. The *Transition* states showed strong connectivity with the *Spinous* states and converged on *Granular*. The existence of distinct trajectories in the spinous cell clusters was previously reported (Reynolds et al., 2021).

Notch pathway gene expression in epidermal cell populations

Next, we investigated which cells express different Notch ligands and receptors (Figure 2). In addition to *DLL1*, *JAG1* and *JAG2* were predominantly expressed in *Basal II* (Figure 2A). The levels of mRNAs for *DLL3* and *DLL4* were almost undetectable (Negri et al., 2019). We also found low levels of Delta-Like Non-Canonical Notch Ligand 1 and 2 (*DLK1* and *DLK2*) in *Basal II* (Figure 2A). In contrast to Notch ligands, Notch receptors were most abundant in the transition, spinous, and granular layer clusters. *NOTCH1* was most abundant in *Transition I*, while *NOTCH2* and *NOTCH3* were more highly expressed in *Spinous* and *Granular*. *NOTCH4* was almost undetectable (Negri et al., 2019).

We also analyzed expression of known Notch targets such as *HES1* and *IRF6* (Figures 2B–2D and S2). The highest expression of *HES1* was in *Transition II*, whereas *IRF6* was more widely expressed (Figures 2B and 2C). This supports a role of Notch signaling in commitment to differentiation (Negri et al., 2019). The enrichment of other pathways related to keratinocyte terminal differentiation and AP1 factors in *Transition II* suggests that this population comprises the cluster most committed to differentiation (Mishra et al., 2017) (Figures S2B and S2C).

Genes encoding proteins that cleave Notch receptors at the plasma membrane to release the Notch intracellular domain (NICD) include *ADAM17*, *ADAM10*, and *PSEN1*, all of which were abundant in *Transition I* and *II* (Figure S3A). In contrast, gamma-secretase subunit *APH1A* was most abundant in *Basal II* (Figure S3A).

The Nedd4 family member of HECT domain E3 ubiquitin ligases NEDD4 (neural precursor cell-expressed developmentally downregulated four/NEDD-1) and NEDD4L (neural precursor cell-expressed developmentally downregulated four-like/NEDD-2) direct Notch receptors to the lysosomes for degradation and are negative regulators of the Notch pathway (Kovall et al., 2017). *NEDD4L* was more abundant than *NEDD4* and was a marker for both *Transition I* and *II* (Figure S3D).

The E3 ubiquitin ligase FBXW7 or FBW7 (F Box and WD Repeat Domain Containing 7) binds directly to NICD in the nucleus, leading to its proteasomal degradation and termination of Notch signaling (Kourtis et al., 2015). We observed particularly high expression of *FBXW7* in *Transition II* (Figures 2B and 2D), the same cluster with the highest levels of the histone deacetylase Sirtuin-1 (SIRT1) (Figures 2B and 2D), which can associate with NICD and counteract the stabilizing effect of acetylation, modulating Notch activity amplitude and duration (Guarani et al., 2011).

Several post-translational modifiers of the Notch pathway exhibited differential expression. *FURIN* was the most abundant gene in *Transition I*, while *POGLUT1* was prominent in *Transition II*. *POGLUT1* was also upregulated in *Basal II*, as were *RFNG* and *LFNG*. *MFNG* was expressed at very low levels (Figures 2B, 2D, and S3B).

In conclusion, the scRNA-seq data support the experimental evidence that Notch signaling regulates the onset of terminal differentiation, with many mediators of the

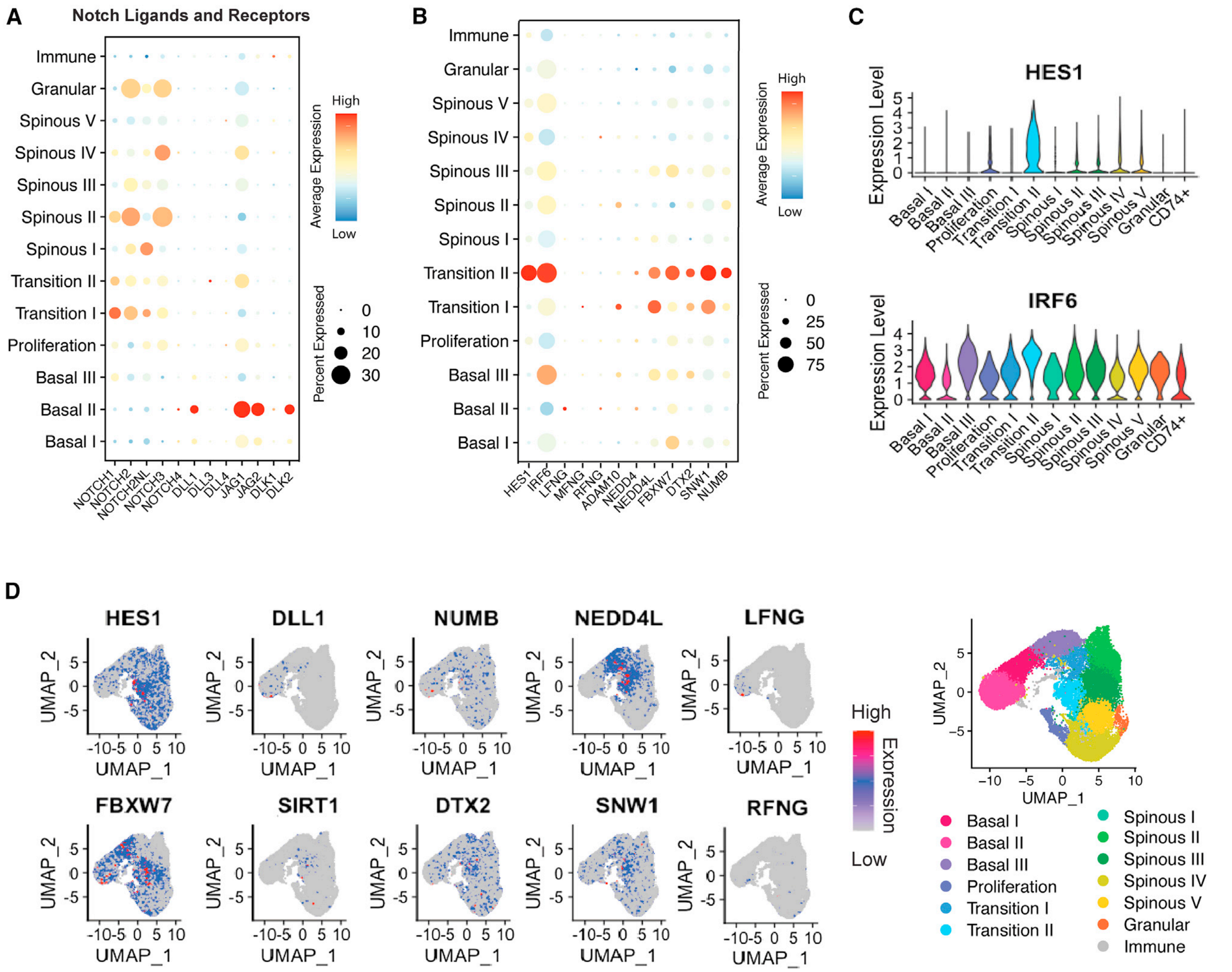

Figure 2. Expression of Notch-related genes in keratinocyte clusters

(A and B) Dot plots showing Notch signaling pathway ligands and receptors (A) and pathway targets and regulators (B).

(C) Violin plots of *HES1* and *IRF6* expression.

(D) UMAP plots of expression distribution of distinct Notch pathway components; location of the 13 clusters (Figure 1A) is included for comparison.

See also Figures S2 and S3.

pathway being upregulated in the *Transition I* and *II* clusters. These findings further emphasize that the stem cell marker DLL1 is expressed at lower levels than other Notch ligands, raising the question of how it plays such a key role in the epidermal basal layer.

Expression of Fringe proteins in the epidermis and cultured keratinocytes

Consistent with the scRNA-seq data (Figure 2B), *LFNG* was more abundant than *RFNG*, and *MFNG* was expressed at very low levels in adult epidermis (Figures 3A and 3B). The same relative expression was observed in human keratinocytes cultured in low-calcium KSFM (keratinocyte serum free medium) medium or on feeders in standard calcium FAD medium (Figure 3C). *MFNG* expression was higher *in vitro* than *in vivo* (Figure 3C). There was higher expression of all *FNG* genes in KSFM than FAD medium, consistent with Fringe expression being more abundant in basal than differentiated cells. Single-molecule fluorescence *in situ* hybridization (smFISH) of *LFNG* transcripts in human epidermis confirmed expression of *LFNG* in basal layer keratinocytes, but it did not distinguish between particular subpopulations of cells (Figure 3D).

We next investigated how *LFNG* and *RFNG* expression was affected when primary keratinocytes (strains km, kn) were exposed to Jagged ligands. We seeded cells on

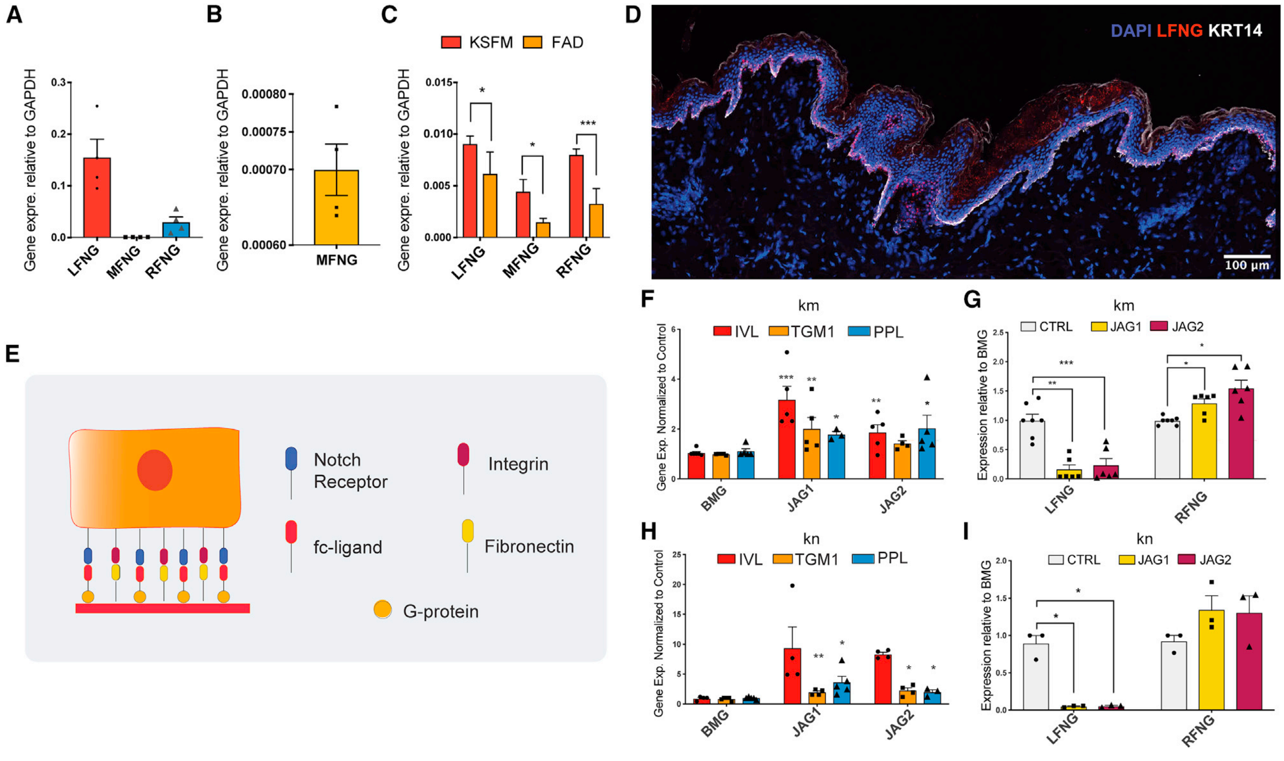

Figure 3. Differential expression of Fringe genes

(A–C) Expression of Fringe genes in human epidermis (A and B) and cultured keratinocytes using qPCR. (C) Average fold change in mRNA abundance (normalized to expression of *RPS18*, *GAPDH*, and *TBP*) compared to control condition. (A–C) Error bars represent standard deviation. Two-tailed, unpaired Student's t test. *p < 0.05, ***p < 0.001. N = 4 samples.

(D) smFISH of adult human skin for *LFNG* (red), counterstained with DAPI (blue) and anti-KRT14 (white). Scale bar, 200 μm.

(E) Schematic of experimental setup for (F)–(I).

(F–I) Expression of differentiation markers (F and H) and *LFNG* and *RFNG* (G and I) in two strains of keratinocytes (KM: km and KN: kn) exposed to recombinant Jagged fc-ligands. Bars represent average fold change compared to control. Each data point is a separate sample. One-way ANOVA with Holm Sidak's multiple comparisons test. *p < 0.05, *p < 0.01, ***p < 0.001. N = 3.

fibronectin in the presence of recombinant Jagged1, Jagged2 or, as a control, beta2 microglobulin (Figure 3E; Negri et al., 2019). Exposing keratinocytes to Jagged1 or Jagged2 led to a significant upregulation of the terminal differentiation marker, *IVL*, with smaller effects on two other differentiation markers, *TGM1* and *PPL* (Figures 3F and 3H). *LFNG* expression was significantly reduced by Jagged1 and Jagged2, whereas *RFNG* was either unaffected or slightly increased (Figures 3G and 3I).

We conclude that in cultured and uncultured human epidermis, *LFNG* is the most abundant Fringe gene and is expressed by basal keratinocytes. When keratinocytes are stimulated to differentiate by exposure to Jagged - ligands, expression of *LFNG* is selectively downregulated.

Overexpression of LFNG increases proliferation and reduces differentiation

To test whether LFNG overexpression in cultured keratinocytes would expand the stem cell compartment, we overexpressed LFNG using a lentiviral vector. As controls, we overexpressed GFP (Figure 4A) or transduced cells with the empty LFNG vector (Figure S4A). Expression of terminal differentiation markers and HES1 was reduced in cells overexpressing LFNG (Figures 4B, 4D, and S4B). Conversely, there was an increase in ΔP63 (Figure 4C).

We detected more Ki67-positive cells in cultures overexpressing LFNG when compared with the controls (Figures 4E and 4F). There was a significant increase in the total number of colonies and the number of large clones formed by keratinocytes overexpressing LFNG (Figures 4G–4I, S4C, and S4D). LFNG overexpression did not affect keratinocyte responsiveness to Notch ligands (Figures 4J and 4K). Nevertheless, there was a reduction in *LFNG* expression in cells exposed to Jagged1 and Jagged2 but not to DLL1 (Figure 4L).

These results suggest that LFNG expression amplifies the effects of DLL1 in epidermal stem cells (Figure 4M).

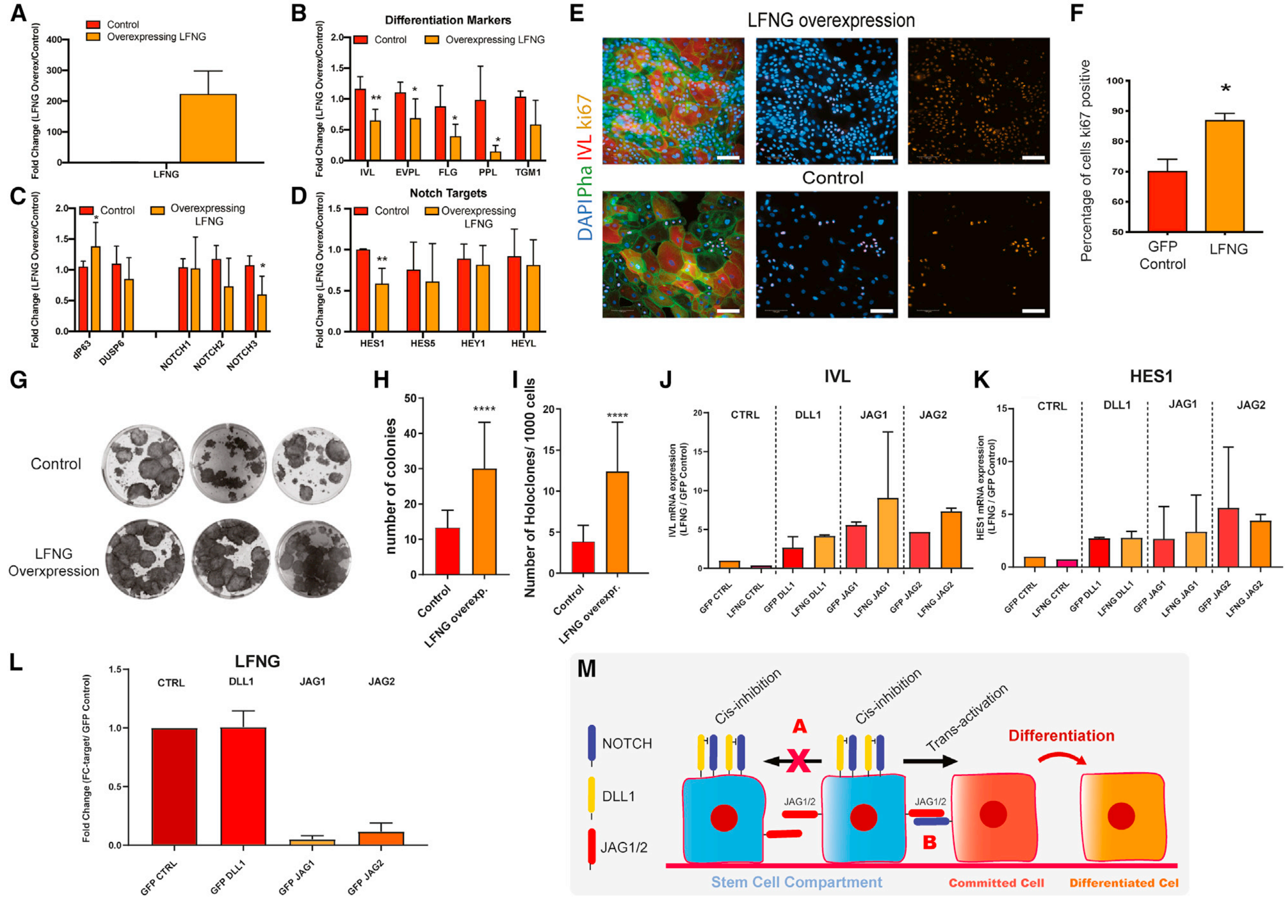

Figure 4. Effects of LFNG overexpression in cultured human keratinocytes

(A–D) Expression profiles of (A) *LFNG*; (B) differentiation markers; (C) Notch receptors, *ΔP63* and *DUSP6*; and (D) Notch targets. Average fold change in mRNA abundance (normalized to expression of *RPS18*, *GAPDH*, and *TBP*) compared to control condition. Error bars represent standard deviation. One-way ANOVA with Holm Sidak's multiple comparisons test. *$p < 0.05$, **$p < 0.01$, ***$p < 0.001$. N = 3 independent samples.

(E and F) Ki67-positive cells determined by immunostaining. (E) Representative confocal images. Scale bar represents 100 μm. (F) Percent Ki67-positive IVL-negative cells. Two-tailed, unpaired Student's t test. *$p < 0.05$. N = 3 independent samples.

(G–I) Effect of LFNG overexpression on colony formation. Three technical replicates from three different lentiviral infections. (G) Representative wells stained with Rhodanile blue. (H) Total colonies per well. (I) Stem cell colonies per well, defined as large colonies.

(J–L) Effect of LFNG or GFP (control) overexpression on response to exogenous DLL1, JAG1, JAG2, or anti-B2MG (control). Standard errors are shown. N = 3 (J and K) and N = 2 (L) independent samples.

(M) Schematic. Epidermal stem cells express LFNG, Jagged1/2, and DLL1. LFNG induces *cis* inhibition based on Notch-DLL1 interaction (A) leaving available Jagged ligands in the membrane. Jagged ligands on the surface of stem cells can only activate cells that do not have LFNG activity (B). The receiving cell activates Notch signaling and thereby initiates terminal differentiation.

See also Figure S4.

DISCUSSION

By reanalyzing published scRNA-seq data, we have been able to test whether stem cell characteristics defined in culture reflect the *in vivo* situation. The data analysis led us to predict that LFNG amplifies the effects of DLL1 in stem cells and thereby design mechanistic experiments.

The clustering data validated the conclusion from earlier experimental studies that there are subpopulations of basal cells that differ in their capacity for self-renewal (Tan et al., 2013). PAGA analysis indicates that *Basal II* cells are the most stem-like state. The scRNA-seq data also allowed identification of new stem cell markers, such as *COL17A1*. Col17A1 protein expression is enriched in basal keratinocytes that lie where the epidermis projects into the underlying dermis (Wang et al., 2020). This is the location of cells with high expression of the stem cell markers *ITGB1*, *CSPG4*, and *CD46* (based on flow cytometry and

immunofluorescence microscopy) and *DLL1* (based on *in situ* hybridization) (Lowell et al., 2000; Tan et al., 2013). One unexpected feature of *Basal I*, *II*, and *III* is that markers that are co-expressed by protein detection methods are differentially expressed at the transcript level. This may reflect differences in protein turnover rates. For example, the turnover rates calculated from cultured breast epithelial cells are approximately 15 h for ITGB1, 23 h for ITGA6, and 45 h for CAV1 (Ly et al., 2018).

In culture, transient upregulation of an interacting network of protein phosphatases acts as an unstable commitment switch between the stem cell and differentiated cell states (Mishra et al., 2017). Unbiased analysis of human epidermis identified two transitional cell populations in which *DUSP6* and *DUSP10* were upregulated. Notch signaling activation was primarily associated with *Transition I* and *II*, on the basis of *HES1*, *IRF6*, *FBXW7*, *NEDD4L*, *DTX2*, and *SIRT1* expression (Estrach et al., 2006; Nguyen et al., 2006). The spatial distribution patterns of Notch ligands and receptors in mouse and human skin (reviewed by Watt et al., 2008) are consistent with the scRNA-seq data.

DLL1 expression protects stem cells from differentiation, mediates stem cell clustering, and instructs neighboring cells to differentiate (Lowell et al., 2000; Negri et al., 2019). However, it is puzzling that DLL1 exerts these effects while being expressed at much lower levels than JAG1 and JAG2, which promote differentiation. scRNA-seq revealed that *LFNG*, like *DLL1*, is most highly expressed in *Basal II*. LFNG increases Notch affinity for DLL1 and decreases affinity for Jagged (Luca et al., 2017; Kakuda and Haltiwanger, 2017). Overexpression of LFNG in cultured human keratinocytes decreased differentiation and promoted colony formation.

Our results suggest that LFNG amplifies the effects of DLL1 in protecting stem cells from undergoing differentiation (Figure 4M). LFNG could act by inducing *cis* inhibition based on Notch-DLL1 interaction, thereby protecting cells from JAG2 signals. *Cis* inhibition between Notch and DLL1 would increase Jagged availability in the plasma membrane (Figure 4M), allowing cells to simultaneously display *cis* inhibition and a sending-signal state based on Jagged. In this way, *Basal II* stem cells could be protected from differentiation and at the same time stimulate neighboring cells to differentiate.

It will be of interest to develop techniques to isolate keratinocyte populations corresponding to *Basal I, II*, and *III*, in order to examine post-translational modification of Notch proteins. Furthermore, by FISH, *LFNG* expression appeared to be uniform in the basal layer, raising the question of to what extent the *Basal II* state is spatially patterned. Additional analysis of the effects of knocking down *LFNG* in keratinocytes and of knockdown and overexpression in skin reconstitution assays will provide more information about the role of Notch signaling in epidermal homeostasis. Extending our bioinformatic analysis to include immune cells and melanocytes may provide new insights into the role of Notch and other signaling pathways in mediating keratinocyte interactions with other cell types.

EXPERIMENTAL PROCEDURES

Resource availability

Corresponding author

Further information and requests for resources and reagents should be directed to and will be fulfilled by the corresponding author, Fiona Watt (fiona.watt@kcl.ac.uk).

Materials availability

All unique/stable reagents generated in this study are available from the corresponding author on completion of a Materials Transfer Agreement.

Data and code availability

The scRNA-seq dataset used in this study has been deposited in ArrayExpress: www.ebi.ac.uk/arrayexpress/experiments/E-MTAB-8142.

scRNA-seq

Quality control metrics for the scRNA-seq data were described previously (Reynolds et al., 2021). Data analysis, dimensionality reduction, cell clustering, and gene profile expression were performed using Seurat's package (v.3.1.5) in R programming language and R-studio (version 1.2.5033) (see supplemental information).

Keratinocyte culture

Neonatal foreskin normal human keratinocytes (strains km and kn) were cultured, as described previously, on a mitotically inactivated feeder layer of J2-3T3 cells in FAD medium (Tan et al., 2013). See supplemental information for differentiation, clonogenicity, and proliferation assays.

RNA extraction and real-time quantitative PCR

Cells were lysed in RLT Lysis Buffer (Qiagen) containing 1% β-mercaptoethanol (Sigma-Aldrich). RNA was isolated using the Qiagen RNeasy mini kit (Qiagen). Complementary DNA synthesis was performed using the SuperScript III Reverse Transcriptase kit (Life Technologies). Real-time quantitative PCR reactions were implemented using TaqManTM probes (Invitrogen) or specifically designed primers (Tables S1 and S2). See supplemental information for real-time quantitative PCR reactions and RNA isolation from tissue.

Functionalized substrates

Six-well plates (Falcon) were coated with human fibronectin (4 $\mu g/cm^2$) and recombinant protein G (Sigma-Aldrich) in PBS and then incubated with Fc-tagged ligand and control recombinant proteins (2.5 $\mu g/cm^2$) (Negri et al., 2019), as described in supplemental information.

Lentivirus transduction

10^6 keratinocytes were seeded per well in collagen type-1 coated six-well plates containing KSFM. After 24 h, 100 μL of lentiviral particles and 5 μg/ml polybrene (Sigma-Aldrich) were added. The following day, the medium was replaced with KSFM containing puromycin (2 μg/ml, Thermo Fisher Scientific). After 48 h, cells were harvested and replated in FAD on feeder cells. The lentivirus constructs were MISSION LentiORF LFNG, MISSION TRC3 ORF GFP Lentivirus Control (Sigma-Aldrich), and pLX307, the commercially available version of pLX_TRC317.

Immunostaining and image analysis

Frozen sections of adult human breast skin were fixed with 4% paraformaldehyde for 20 min and stored at −80°C prior to staining. Sections were blocked and incubated overnight with primary antibodies: anti-keratin14 (Biolegend, 906001) and anti-CD74 (Abcam, ab64772). Slides were washed three times with PBS and labeled with DAPI and secondary antibodies for 1 h (A21206, A32570, A2287, Thermo Fisher Scientific). Samples were mounted in ProLong 394 Gold anti-fade (Thermo Fisher Scientific).

RNAscope

Human skin samples (obtained with informed consent, subject to both institutional and external research ethics council (REC) review (REC reference 19/NE/0063) were embedded in Tissue-Tek O.C.T. (Life Technologies, Waltham, MA, USA) and stored at −80°C. 10-μm sections were cut with a Thermo Cryostar Nx70 (Thermo Fisher Scientific, Waltham, MA, USA) and placed on SuperFrost Plus glass slides (Thermo Fisher Scientific, ref J2800AMN2). Sections were labeled using the RNAscope Multiplex Fluorescent Detection Kit v2 (ACDBio, Newark, California, USA, cat. no. 323100). See supplemental information for more information.

Statistical tests and graphing

Statistical analysis was performed using GraphPad Prism (version 7.0). Data are shown as the mean and standard error of the mean unless otherwise stated. Data were analyzed using Student's t tests, one or two-way ANOVA with Tukey's multiple comparison post test. $p < 0.05$ was considered significant. Graphs were obtained using GraphPad Prism 7 or ggplot2 R package version 3.1.0 (https://ggplot2.tidyverse.org).

SUPPLEMENTAL INFORMATION

Supplemental information can be found online at https://doi.org/10.1016/j.stemcr.2023.09.007.

ACKNOWLEDGMENTS

We gratefully acknowledge the advice and practical contributions of Simon Broad, Arsham Ghahramani, Eamonn Morrison, Gernot Walko, and Vasiliki Salameti. This work was supported by grants to FMW from the UK Medical Research Council (MR/PO18823/1) and the Wellcome Trust (206439/Z/17/Z). VAN is the recipient of a National Council for Scientific and Technological Development-Brazil (CNPq) doctoral scholarship. BL and SZ received support from the Danish National Research Foundation (DNRF135). MH is funded by Wellcome (WT107931/Z/15/Z), The Lister Institute for Preventive Medicine and NIHR and Newcastle-Biomedical Research Centre. MH and FMW acknowledge funding from the Wellcome Human Cell Atlas Strategic Science Support (WT211276/Z/18/Z). FMW also acknowledges funding from the Department of Health via the National Institute for Health Research comprehensive Biomedical Research Centre award to Guy's & St Thomas' National Health Service Foundation Trust in partnership with King's College London and King's College Hospital NHS Foundation Trust.

AUTHOR CONTRIBUTIONS

Conceptualization: V.A.N. and F.M.W.; data curation: V.A.N., S.A., G.R., and M.H.; formal analysis: V.A.N. and G.R.; funding acquisition: V.A.N., M.H., and F.M.W.; investigation: V.A.N. and B.L.; methodology: V.A.N., B.L., S.Z., C.G., and C.P.; project administration: F.M.W.; software: V.A.N., S.A., and G.R.; supervision: F.M.W.; validation: V.A.N., B.L., S.Z., and C.G.; writing – first draft: V.A.N. and F.M.W.; writing – review and editing: all.

DECLARATION OF INTERESTS

F.M.W. is a member of the *Stem Cell Reports* editorial board and receives research funding from POLA.

Received: December 26, 2021
Revised: September 14, 2023
Accepted: September 14, 2023
Published: October 12, 2023

REFERENCES

Blanpain, C., Lowry, W.E., Pasolli, H.A., and Fuchs, E. (2006). Canonical Notch signaling functions as a commitment switch in the epidermal lineage. Genes Dev. *20*, 3022–3035.

Cheng, J.B., Sedgewick, A.J., Finnegan, A.I., Harirchian, P., Lee, J., Kwon, S., Fassett, M.S., Golovato, J., Gray, M., Ghadially, R., et al. (2018). Transcriptional programming of normal and inflamed human epidermis at single-cell resolution. Cell Rep. *25*, 871–883.

Estrach, S., Ambler, C.A., Lo Celso, C., Hozumi, K., and Watt, F.M. (2006). Jagged 1 is a beta-catenin target gene required for ectopic hair follicle formation in adult epidermis. Development *133*, 4427–4438.

Estrach, S., Cordes, R., Hozumi, K., Gossler, A., and Watt, F.M. (2008). Role of the Notch ligand Delta1 in embryonic and adult mouse epidermis. J. Invest. Dermatol. *128*, 825–832.

Guarani, V., Deflorian, G., Franco, C.A., Krüger, M., Phng, L.-K., Bentley, K., Toussaint, L., Dequiedt, F., Mostoslavsky, R., Schmidt, M.H.H., et al. (2011). Acetylation-dependent regulation of endothelial Notch signalling by the SIRT1 deacetylase. Nature *473*, 234–238.

Irvine, K.D. (2008). A Notch sweeter. Cell *132*, 177–179.

Kakuda, S., and Haltiwanger, R.S. (2017). Deciphering the Fringe-mediated Notch code: identification of activating and inhibiting sites allowing discrimination between ligands. Dev. Cell *40*, 193–201.

Kourtis, N., Strikoudis, A., and Aifantis, I. (2015). Emerging roles for the FBXW7 ubiquitin ligase in leukemia and beyond. Curr. Opin. Cell Biol. *37*, 28–34.

Kovall, R.A., Gebelein, B., Sprinzak, D., and Kopan, R. (2017). The canonical Notch signaling pathway: structural and biochemical insights into shape, sugar, and force. Dev. Cell *41*, 228–241.

LeBon, L., Lee, T.V., Sprinzak, D., Jafar-Nejad, H., and Elowitz, M.B. (2014). Fringe proteins modulate Notch-ligand cis and trans interactions to specify signaling states. Elife *3*, e02950.

Lowell, S., Jones, P., Le Roux, I., Dunne, J., and Watt, F.M. (2000). Stimulation of human epidermal differentiation by delta-notch signalling at the boundaries of stem-cell clusters. Curr. Biol. *10*, 491–500.

Luca, V.C., Kim, B.C., Ge, C., Kakuda, S., Wu, D., Roein-Peikar, M., Haltiwanger, R.S., Zhu, C., Ha, T., and Garcia, K.C. (2017). Notch--Jagged complex structure implicates a catch bond in tuning ligand sensitivity. Science *355*, 1320–1324.

Ly, T., Endo, A., Brenes, A., Gierlinski, M., Afzal, V., Pawellek, A., and Lamond, A.I. (2018). Proteome-wide analysis of protein abundance and turnover remodelling during oncogenic transformation of human breast epithelial cells. Wellcome Open Res. *3*, 51.

Mishra, A., Oulès, B., Pisco, A.O., Ly, T., Liakath-Ali, K., Walko, G., Viswanathan, P., Tihy, M., Nijjher, J., Dunn, S.-J., et al. (2017). A protein phosphatase network controls the temporal and spatial dynamics of differentiation commitment in human epidermis. Elife *6*, e27356.

Negri, V.A., Logtenberg, M.E.W., Renz, L.M., Oules, B., Walko, G., and Watt, F.M. (2019). Delta-like 1-mediated cis-inhibition of Jagged1/2 signalling inhibits differentiation of human epidermal cells in culture. Sci. Rep. *9*, 10825.

Negri, V.A, and Watt, F.M (2022). Understanding Human Epidermal Stem Cells at Single-Cell Resolution. J Invest Dermatol *142*, 2061–2067.

Nguyen, B.C., Lefort, K., Mandinova, A., Antonini, D., Devgan, V., Della Gatta, G., Koster, M.I., Zhang, Z., Wang, J., and Di Vignano, A.T. (2006). Cross-regulation between Notch and p63 in keratinocyte commitment to differentiation. Genes Dev. *20*, 1028–1042.

Rangarajan, A., Talora, C., Okuyama, R., Nicolas, M., Mammucari, C., Oh, H., Aster, J.C., Krishna, S., Metzger, D., Chambon, P., et al. (2001). Notch signaling is a direct determinant of keratinocyte growth arrest and entry into differentiation. EMBO J. *20*, 3427–3436.

Reynolds, G., Vegh, P., Fletcher, J., Poyner, E.F.M., Stephenson, E., Goh, I., Botting, R.A., Huang, N., Olabi, B., Dubois, A., et al. (2021). Developmental cell programs are co-opted in inflammatory skin disease. Science *371*, eaba6500.

Stanley, P., and Okajima, T. (2010). Roles of glycosylation in Notch signaling. Curr. Top. Dev. Biol. *92*, 131–164.

Su, H., Na, N., Zhang, X., and Zhao, Y. (2017). The biological function and significance of CD74 in immune diseases. Inflamm. Res. *66*, 209–216.

Tan, D.W.M., Jensen, K.B., Trotter, M.W.B., Connelly, J.T., Broad, S., and Watt, F.M. (2013). Single-cell gene expression profiling reveals functional heterogeneity of undifferentiated human epidermal cells. Development *140*, 1433–1444.

Wang, S., Drummond, M.L., Guerrero-Juarez, C.F., Tarapore, E., MacLean, A.L., Stabell, A.R., Wu, S.C., Gutierrez, G., That, B.T., Benavente, C.A., et al. (2020). Single cell transcriptomics of human epidermis identifies basal stem cell transition states. Nat. Commun. *11*, 4239.

Watt, F.M. (2014). Mammalian skin cell biology: at the interface between laboratory and clinic. Science *346*, 937–940.

Watt, F.M., Estrach, S., and Ambler, C.A. (2008). Epidermal Notch signalling: differentiation, cancer and adhesion. Curr. Opin. Cell Biol. *20*, 171–179.

Zhao, S., Zhang, L., Xiang, S., Hu, Y., Wu, Z., and Shen, J. (2022). Gnawing between cells and cells in the immune system: friend or foe? A review of trogocytosis. Front. Immunol. *13*, 791006.

Stem Cell Reports, Volume *18*

Supplemental Information

Single-cell RNA sequencing of human epidermis identifies Lunatic fringe as a novel regulator of the stem cell compartment

Victor Augusti Negri, Blaise Louis, Sebastiaan Zijl, Clarisse Ganier, Christina Philippeos, Shahnawaz Ali, Gary Reynolds, Muzlifah Haniffa, and Fiona M. Watt

Supplementary Material

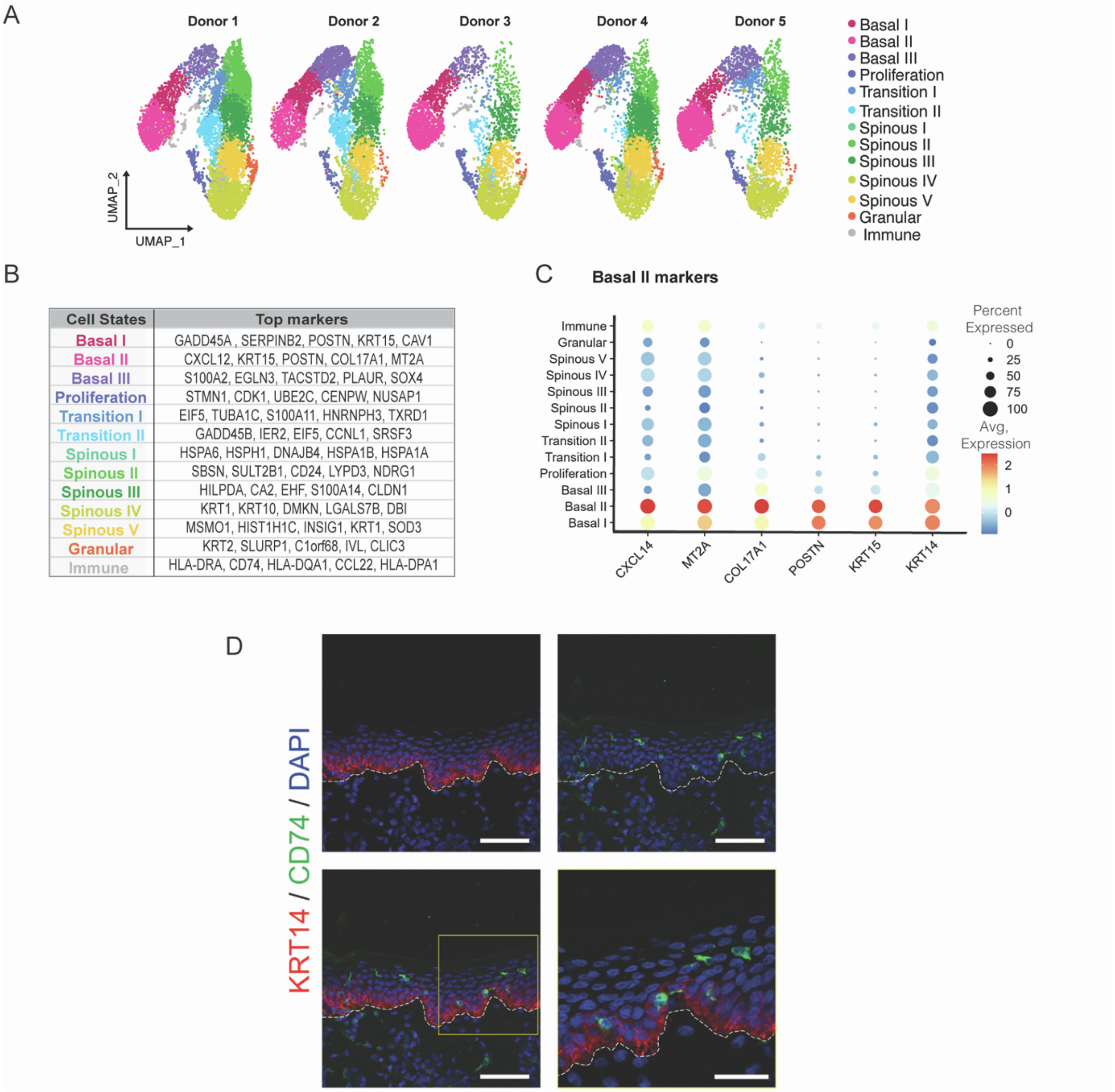

Cell States	Top markers
Basal I	GADD45A , SERPINB2, POSTN, KRT15, CAV1
Basal II	CXCL12, KRT15, POSTN, COL17A1, MT2A
Basal III	S100A2, EGLN3, TACSTD2, PLAUR, SOX4
Proliferation	STMN1, CDK1, UBE2C, CENPW, NUSAP1
Transition I	EIF5, TUBA1C, S100A11, HNRNPH3, TXRD1
Transition II	GADD45B, IER2, EIF5, CCNL1, SRSF3
Spinous I	HSPA6, HSPH1, DNAJB4, HSPA1B, HSPA1A
Spinous II	SBSN, SULT2B1, CD24, LYPD3, NDRG1
Spinous III	HILPDA, CA2, EHF, S100A14, CLDN1
Spinous IV	KRT1, KRT10, DMKN, LGALS7B, DBI
Spinous V	MSMO1, HIST1H1C, INSIG1, KRT1, SOD3
Granular	KRT2, SLURP1, C1orf68, IVL, CLIC3
Immune	HLA-DRA, CD74, HLA-DQA1, CCL22, HLA-DPA1

Figure S1 related to Figure 1. A*:* UMAP plot showing the 13 distinct cell states found in the 5 distinct donors used for this study B*:* Top 5 genes differentially expressed in each cell state. For this analysis we applied Wilcoxon rank sum test and considered genes as differentially expressed when the average log fold change test (avg_logFC) was higher than 0.5 and p-value for multiple testing (Bonferroni) was lower than 0.001. C: DotPlot showing scaled normalized expression of selected genes differentially expressed in *Basal II* cluster*.* D: Adult human skin labelled with antibodies to CD74 (green) and Krt14 (red) with DAPI nuclear counterstain (blue). Scale bars: 60µm, 30µm (high magnification of boxed area).

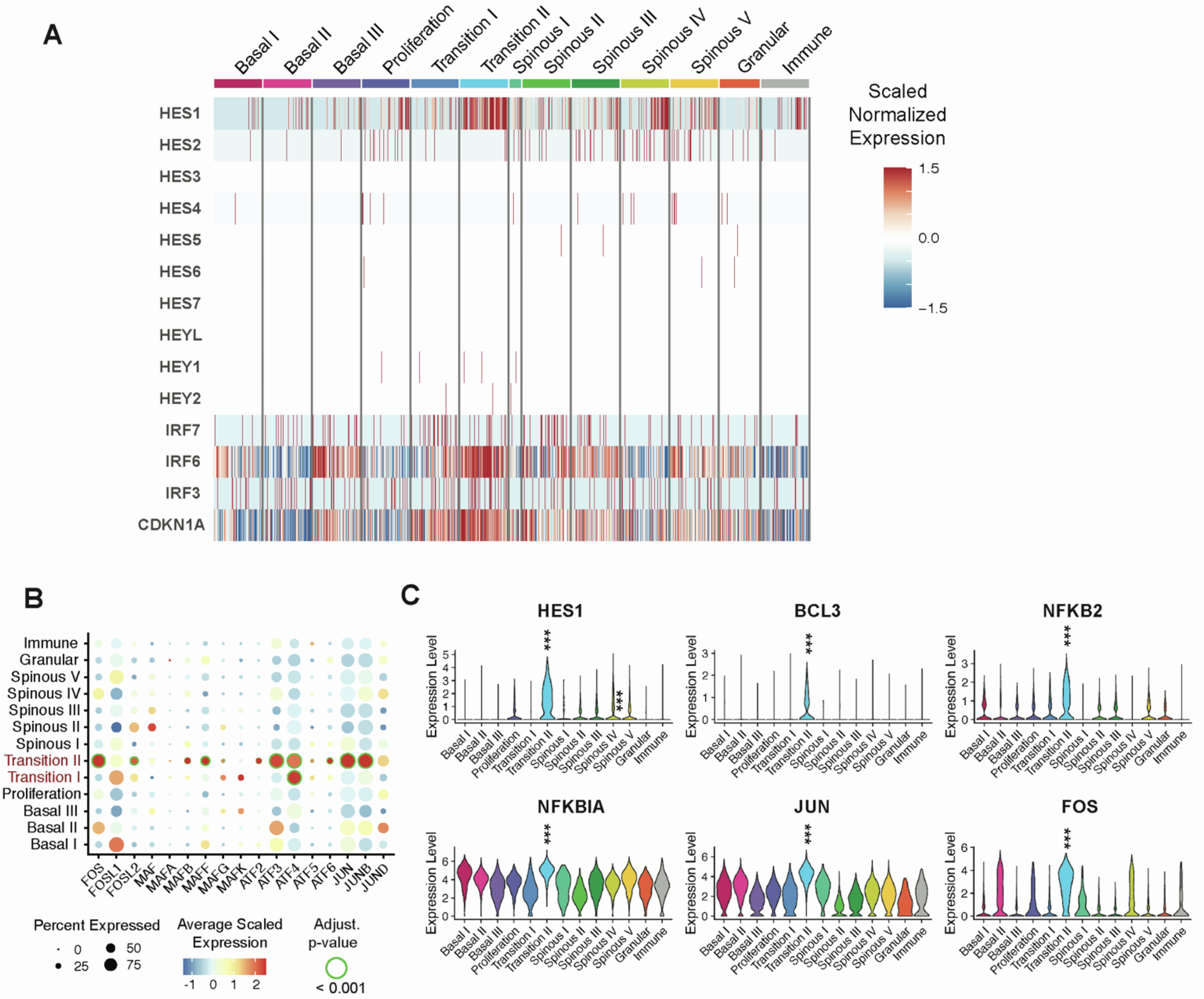

Figure S2 related to Figure 2. A: Heatmap plot showing scaled expression values of known Notch pathway target genes in epidermal cells (n = 5, 27138 cells). B: Dot plot showing expression distribution of AP1 factors in distinct epidermal cell states. Green circles: adjusted p-value < 0.001 (Bonferroni Correction). C: Violin plots showing normalized expression distribution of a selection of markers in the distinct cell states. Adjusted p-value <0.001 (***).

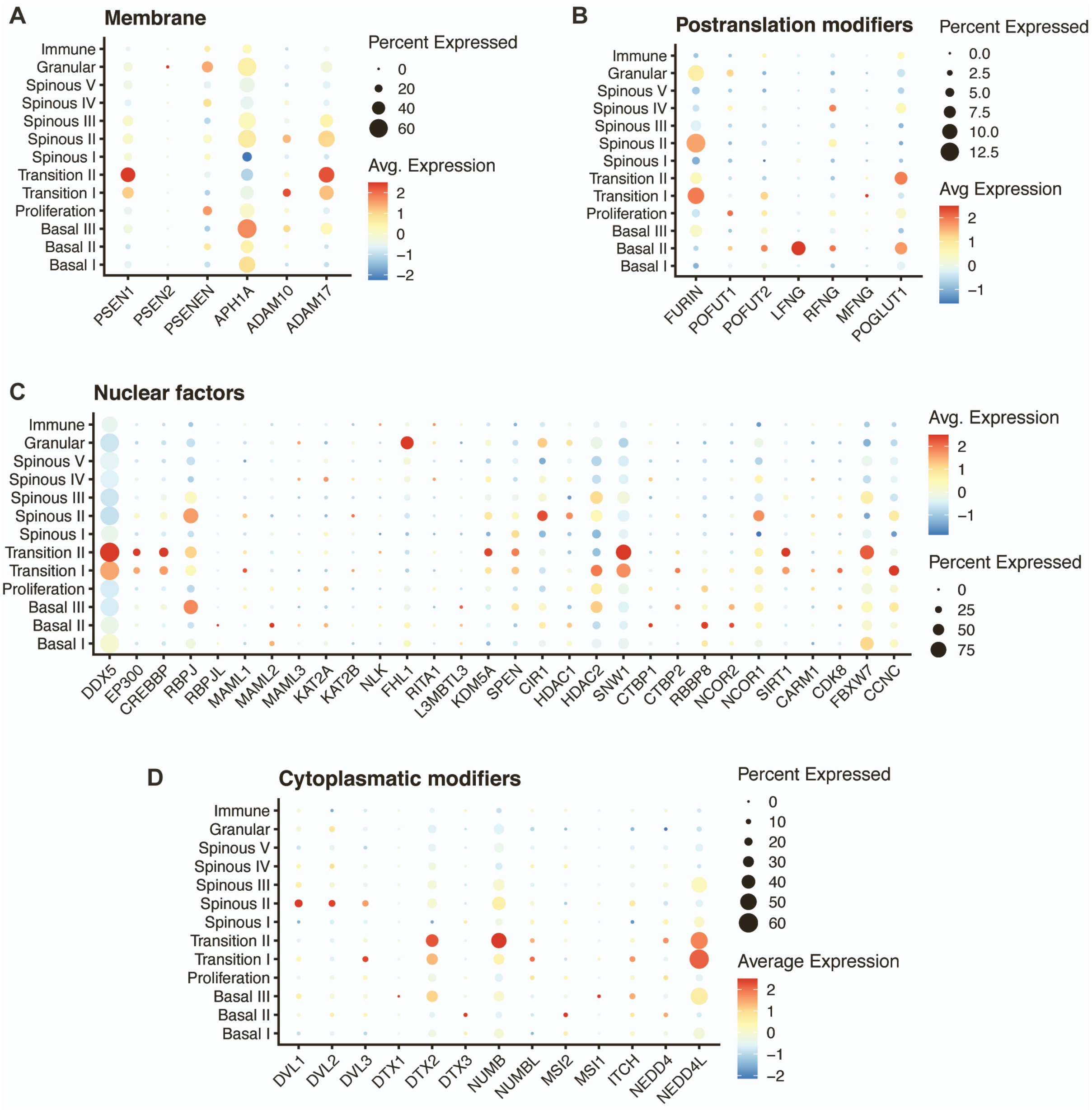

Figure S3 related to Figure 2. Dot plots showing distribution of the normalized expression of Notch signalling pathway regulators. A: Notch associated genes encoding proteins present in the plasma membrane; B: genes associated with Notch receptor and ligand posttranslational modifications; C: nuclear Notch regulatory factors; D: Notch cytoplasmic regulatory factors.

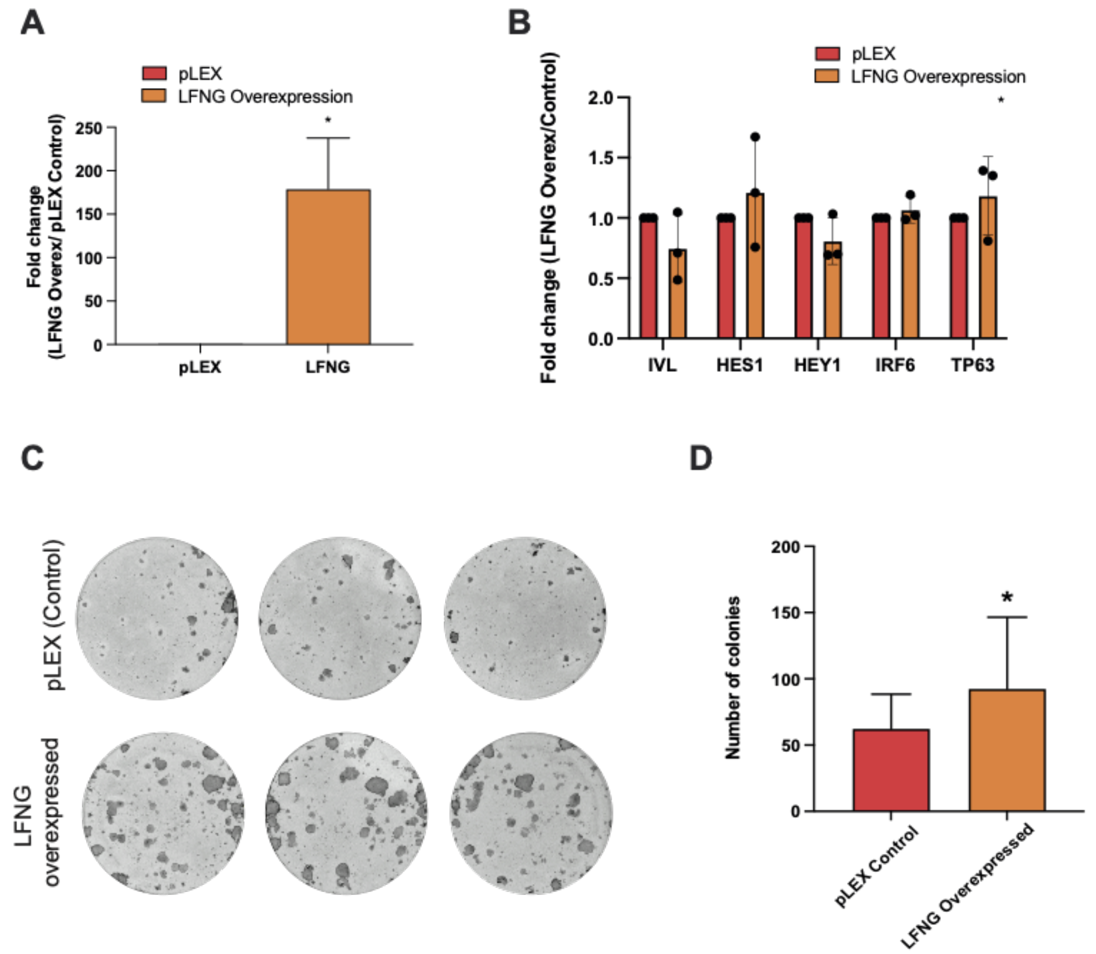

Figure S4 related to Figure 4. LFNG overexpression in primary human keratinocytes. A: Expression levels by qPCR of LFNG in LFNG overexpressed keratinocytes and pLEX controls. B: Expression levels of Notch targets and terminal differentiation markers in LFNG overexpressing keratinocytes and control (pLEX). C-D: Effect of LFNG overexpression on colony formation. 1000 cells were seeded per well. Assays were performed on three technical replicates from three different lentiviral infections. C: Representative wells stained with 1% Rhodanile Blue. D: Total number of colonies per well. P value of 0.05 or less was considered statistically significant (*). N=3.

Supplemental Table 1 - TaqMan probes used for the qPCR reactions (related to Experimental Procedures)

Gene Name	Gene Symbol	TaqMan Assay ID
Delta-Like Ligand 1	DLL1	Hs.379912_m1
Glyceraldehyde-3-phosphate dehydrogenase	GAPDH	Hs02758991_g1
Hes family bHLH transcription factor 1	HES1	Hs00172878_m1
Hes family bHLH transcription factor 2	HES2	Hs01021800_g1
Hes family bHLH transcription factor 4	HES4	Hs00970270_g1
Hes family bHLH transcription factor 5	HES5	Hs01387463_g1
Hes family bHLH transcription factor 6	HES6	Hs05043218_s1
Hes family bHLH transcription factor 7	HES7	Hs00261517_m1
Hes related family bHLH transcription factor with YRPW motif 1	HEY1	Hs01114113_m1
Interferon regulatory factor 6	IRF6	Hs01062178_m1
Involucrin	IVL	Hs00846307_s1
Jagged 1	JAG1	Hs01070032_m1
Jagged 2	JAG2	Hs00171432_m1
Lunatic Fringe	LFNG	Hs00385436_g1
Manic Fringe	MFNG	Hs00159117_m1
Radical Fringe	RFNG	Hs01357010_g1
Ribossomal protein S18	RPS18	Hs01375212_g1
TATA-box binding protein	TBP	Hs00427620_m1
Tumor protein p63	TP63	Hs00978340_m1
Hes related family bHLH transcription factor with YRPW motif 2	HEY2	Hs01012057_m1
Hes related family bHLH transcription factor with YRPW motif-like	HEYL	Hs01113778_m1

Supplemental Table 2 - Primer pairs used in SYBR green RT qPCR reactions (related to Experimental Procedures)

Gene Name	Gene Symbol	Primer Forward	Primer Reverse
Hes family bHLH transcription factor 1	HES1	AAAAATTCCTCGTCCCCGGT	ATGCCGCGAGCTATCTTTCT
Ribossomal protein S18	18sRNA	GCAATTATTCCCCATGAACG	GGCCTCACTAAACCATCCAA
DeltaNp63 - p63 isoform	dNtp63	TCCTCAGGGAGCTGTTATCC	TGACTAGGAGGGGCAATCTG
Dual Specificity Phosphatase 6	DUSP6	ACCTGGAAGGTGGCTTCAGT	CTCGGTCAAGGTCAGACTCG
Hes related family bHLH transc. factor with YRPW motif1	HEY1	GTTCGGCTCTAGGTTCCATGT	CGTCGGCGCTTCTCAATTATTC
Interferon regulatory factor 6	IRF6	GCTCTCTCCCAATGACCTGGA	CCATGACGTCCAGCAGCTTGCTA
Integrin subunit alfa 6	ITGA6	CGCTGGGATCTTGATGCTTGCT	TGAGCATGGATCTCAGCCTTGTGA
Integrin subunit beta 1	ITGB1	GACGCCGCGCGGAAAAGATG	ACCACCCACAATTTGGCCCTGC
Involucrin	IVL	TCCTCCAGTCAATACCCATC	CAGCAGTCATGTGCTTTTCCT
Notch Receptor 1	NOTCH1	TCCACCAGTTTGAATGGTCA	AGCTCATCATCTGGGACAGG
Notch Receptor 2	NOTCH2	GATCACCCGAATGGCTATGAAT	GGGGTCACAGTTGTCAATGTT
Notch Receptor 3	NOTCH3	TGGCGACCTCACTTACGACT	CACTGGCAGTTATAGGTGTTGAC
Notch Receptor 4	NOTCH4	TGTGAACGTGATGTCAACGAG	ACAGTCTGGGCCTATGAAACC
NUMB, endocytic adaptor protein	NUMB	GGCATACAGAGGTTCCTACA	TGCTCCTTTGACCGCTAC
Periplakin	PPL	GCAGAGTGACCTGGCTCGGCT	GCCGCATCCGCCTCTAGCAC
TATA-box binding protein	TBP	GTGACCCAGCATCACTGTTTC	GAGCATCTCCAGCACACTCT
Transglutaminase 1	TGM1	GCACCACACAGACGAGTATGA	GGTGATGCGATCAGAGGAT

Supplemental Experimental Procedures (related to Experimental Procedures)

scRNAseq analysis

To reconstruct the keratinocyte differentiation programme we converted the Seurat object in a SCANPY H5AD file using the SeuratDisk package (Stuart et al., 2019; Hoffman, 2021) and applied Partition-based approximate graph abstraction (PAGA) using Scanpy v1.9.1 (sc.tl.paga) on python version 3.9.5 (Wolf et al., 2018).

Raw counts were normalized using NormalizedData (scalefactor = 1000, method = LogNormalize) and scaled using ScaleData. Principal component analysis (PCA) was performed using Seurat's RunPCA function and considering the 2000 most variable genes (obtained with FindVariableFeatures Seurat's function). Uniform manifold approximation and projection (UMAP) dimensional reduction was obtained based on the 30 first principal components (Pei et al., 2018). Next, we determined the k-nearest neighbor graph using the FindNeighbors function. Cell clustering was performed by Louvain's clustering using FindClusters function (resolution = 0.5). To label and identify the clusters, markers were obtained with FindAllMarkers applying the non-parametric Wilcoxon Rank Sum test. Distinct cell states were annotated manually based on known markers and the most significantly expressed genes in each cluster (adjusted p-value adjusted < 0.001, Bonferroni correction) (Figure S1). Application of DoubletFinder (McGinnis et al., 2019) ruled out the possibility that the Immune cell state consisted of cell doublets. Differential gene expression was determined using FindMarkers and the non-parametric Wilcoxon Rank Sum test. Genes with a log-fold change > 0.5 and adjusted P value (Bonferroni correction) < 0.001 were considered differentially expressed.

Keratinocyte culture

Complete FAD medium comprises 1-part Ham's F12 medium, three parts DMEM and 1.8×10^{-4} M adenine (Life Technologies), 10 % foetal bovine serum (Gibco), 0.5 µg/ml, hydrocortisone (Thermo Fisher Scientific), 5 µg/ml insulin (Sigma Aldrich), 10^{-10}M cholera toxin (Enzo-Life Science), 100 µg/ml streptomycin (Life Technologies), 100U/ml penicillin (Life Technologies), 10 ng/ml epidermal growth factor (EGF), 450 µg/ml glutamine (Life Technologies) n some experiments keratinocytes were transferred, feeder-free, to collagen type-1 (20µg/mL) coated surfaces in keratinocyte serum-free medium (KSFM, Thermo Fisher Scientific) supplemented with bovine pituitary extract (30µg/ml) and epidermal growth factor (EGF, 0.2 ng/ml, Thermo Fisher Scientific) (complete KSFM). To stimulate

differentiation prior to RNA isolation 1x10^5 cells per plated per well of 12 well-plates in complete KSFM medium for 24 hours and then transferred to FAD medium for 48 or 72 hours.

For clonogenicity assays 10^3 cells were plated per well of 6-well plates containing J2-3T3 feeders and then fixed and stained with Rhodanile Blue as described previously (Mishra et al., 2017). Colony formation was quantitated using a Molecular Imager Gel Doc XR+ (Bio-Rad) and ImageJ software.

For proliferation evaluation cells were seeded for 48 hours in FAD in a 96 well plate and labelled with anti-Ki67, DAPI (Invitrogen) and phalloidin (Alexa FluorTM 647 Phalloidin, Thermo Fisher Scientific). Plates were imaged using an Operetta (Perkin-Elmer), and images were quantified using the Harmony® high-content analysis software package (Perkin- Elmer). In the analysis pipeline (Louis et al., 2022) nuclei were identified by DAPI staining and incomplete cells at the borders of the images were excluded. A staining intensity threshold was used to identify Ki67 positive nuclear staining relative to background.

Real-Time Quantitative PCR reactions and RNA isolation from tissue

RT-qPCR reactions were performed with the SYBRT^M Green PCR Master Mix (Thermo Fisher Scientific) or on a CFX 384 Touch RT- qPCR machine (Bio-Rad). Ct values were normalized to the ct values of 18S, GAPDH and TBP (housekeeping genes). Expression values were obtained applying the -ΔΔCT method (Livak and Schmittgen, 2001).

To isolate RNA from human skin (surgical waste, obtained under NHS Research Ethics approval 19/NE/0063), the tissue was minced with a scalpel and incubated for 12 hours at 4°C in 0.2% Dispase (Corning) prior to separating the epidermis from the dermis with tweezers. The skin fragments were transferred to tubes containing TrypLE (Thermo Fisher Scientific) for 10 minutes. Undigested material was discarded following centrifugation at 150 x g for 5 minutes. After two washes with PBS, total RNA was extracted using the PureLink-RNA Mini-Kit (Thermo Fisher Scientific).

Functionalised substrates

Human recombinant proteins were as follows: Jagged 2 Fc Chimera, CF (RnD systems), Jagged 1 Fc Chimera (RnD systems), DLL1 Fc chimera (Adipogen). Beta-2-microglobulin (clone B2M-01, IgG2a) antibody was obtained from Abcam. Human keratinocytes were trypsinized with the addition of DAPT (10µM final, gama-secretase inhibitor, Sigma-Aldrich). Cells (5x10^4) were added to the plates containing KSFM medium supplemented with DAPT (10µM, Sigma Aldrich). After 3 hours, the medium was removed, and KSFM medium supplemented with calcium ($CaCl_2$, 1.2mM) was added.

RNAscope

RNA integrity was confirmed with housekeeping control probes (high (UBC), medium (PPIB) and low (POLR2A) expression). A 20zz probe of human LFNG mRNA was designed by the ACD Probe Design Team targeting 296-1480 of NM_001040167.2 of the LFNG Sequence in FASTA format. This design targets all 4 transcript variants on NCBI. The Opal 520 Reagent Pack (Akoya Biosciences, Marlborough, Massachusetts, USA, ref : FP1487001KT) was used at a dilution of 1:1,000 for the fluorophore step to develop the channel associated with the LFNG probe. Labelling was followed by three 5 minute washes in PBS containing Tween (0.1%), followed by incubation in blocking buffer containing 10% serum, 0.2% fish skin gelatin, 0.1% BSA, and 0.5% Tween-20 (all Sigma-Aldrich, St. Louis, MO, USA) in PBS. Sections were labelled with Chicken IgY anti-Human Keratin 14 primary antibody (Biolegend, Inc, San Diego, CA, USA, ref 906001) in blocking buffer overnight at 4°C. Sections were washed with PBS and then labelled with Goat anti-Chicken IgY (H+L) Secondary Antibody conjugated with Alexa Fluor™ 647 (Thermo Fisher Scientific, ref A21449) for 1 hour at room temperature, followed by PBS washes. Nuclei were counterstained with 4′,6-diamidino-2-phenylindole and mounted using ProLong Gold Antifade Mountant (Thermo Fisher, cat. no. P36930). Slides were imaged with a Nikon A1 upright confocal microscope (Nikon, Tokyo, Japan) using a 20 dry lens and were processed using Fiji software.

Supplemental Acknowledgements (related to Acknowledgements)

This work was supported by grants to FMW from the UK Medical Research Council (MR/PO18823/1) and the Wellcome Trust (206439/Z/17/Z). VAN is the recipient of a National Council for Scientific and Technological Development-Brazil (CNPq) doctoral scholarship. BL and SZ received support from the Danish National Research Foundation (DNRF135). MH is funded by Wellcome (WT107931/Z/15/Z), The Lister Institute for Preventive Medicine and NIHR and Newcastle-Biomedical Research Centre. MH and FMW acknowledge funding from the Wellcome Human Cell Atlas Strategic Science Support (WT211276/Z/18/Z). FMW also acknowledges funding from the Department of Health via the National Institute for Health Research comprehensive Biomedical Research Centre award to Guy's & St Thomas' National Health Service Foundation Trust in partnership with King's College London and King's College Hospital NHS Foundation Trust.

Supplemental References (related to Supplemental Experimental Procedures)

Hoffman, P. (2021) SeuratDisk: interfaces for HDF5-based single cell file formats.

Livak, K. J. and Schmittgen, T. D. (2001) Analysis of relative gene expression data using real-

Louis, B., Tewary, M., Bremer, A.W., Philippeos, C., Negri, V.A., Zijl, S., Gartner, Z.J., Schaffer, D.V., and Watt, F.M. (2022) A reductionist approach to determine the effect of cell-cell contact on human epidermal stem cell differentiation. Acta Biomater. *150*, 265-276.

McGinnis, C.S., Murrow, L.M., and Gartner, Z.J. (2019). DoubletFinder: doublet detection in single-cell RNA sequencing data using artificial nearest neighbors. Cell Syst. *8*, 329-337.

Stuart, T., Butler, A., Hoffman, P., Hafemeister, C., Papalexi, E., Mauck, W.M. 3rd, Hao, Y., Stoeckius, M., Smibert, P., and Satija, R. (2019) Comprehensive integration of single-cell data. Cell *177*, 1888-1902.

Stuart, T., Butler, A., Hoffman, P., Hafemeister, C., Papalexi, E., Mauck, W.M. 3rd, Hao, Y., Stoeckius, M., Smibert, P., and Satija, R. (2019) Comprehensive integration of single-cell data. Cell *177*, 1888-1902.

Wolf, F. A., Angerer P., Theis, F.G. (2018) SCANPY: Large-scale single-cell gene expression data analysis. Genome Biol. *19,* 15.
